# Identification and validation of tissue or ctDNA PTPRD phosphatase domain deleterious mutations as prognostic and predictive biomarkers for immune checkpoint inhibitors in non-squamous NSCLC

**DOI:** 10.1186/s12916-021-02075-5

**Published:** 2021-10-07

**Authors:** Yiting Sun, Jianchun Duan, Wenfeng Fang, Zhijie Wang, Xinyang Du, Xin Wang, Chengcheng Li, Shangli Cai, Jie Zhao, Sini Li, Li Zhang, Hua Bai, Jie Wang

**Affiliations:** 1 State Key Laboratory of Molecular Oncology, Department of Medical Oncology, National Cancer Center/National Clinical Research Center for Cancer/Cancer Hospital, Chinese Academy of Medical Sciences & Peking Union Medical College, 17 Pan-jia-yuan South Lane, Chaoyang District, Beijing, 100021 China; 2 Medical Oncology, Sun Yat-sen University Cancer Center, 651# East Dong Feng Road, Guangzhou, 510060 Guangdong China; 3 Thoracic Surgery Department, National Cancer Center/National Clinical Research Center for Cancer/Cancer Hospital, Chinese Academy of Medical Sciences and Peking Union Medical College, Beijing, China; 4 The Medical Department, Burning Rock Biotech, Guangzhou, China

**Keywords:** Biomarker, PTPRD, Immune checkpoint inhibitor, Non-small cell lung cancer

## Abstract

**Background:**

With the revolutionary progress of immune checkpoint inhibitors (ICIs) achieved in non-small cell lung cancers (NSCLC), identifying patients benefiting from ICIs becomes critical and urgent. The associations of genomic alterations in protein tyrosine phosphatase receptor-type (PTPRs) and ICIs responses are unknown.

**Methods:**

Whole-exome sequencing (WES) of 73 advanced NSCLC tumors sampled before anti-PD-(L)1 therapy was carried out with corresponding clinical data collected as a discovery cohort to find the associations of PTPR mutations and ICI responses. Three validation cohorts consolidated by 7 public cohorts of 1920 NSCLC patients with WES or target sequencing data of tumor tissue-derived DNA or circulating tumor DNA (ctDNA) and relevant clinical data were applied as validation cohorts. The lung adenocarcinoma (LUAD) cohort (*n*=586) in The Cancer Genome Atlas (TCGA) database was used for analyzing the potential anti-tumor immunologic mechanisms.

**Results:**

With the highest mutation frequency among all PTPRs, PTPRD mutations in non-squamous NSCLC (ns-NSCLC) were linked to longer progression-free survivals (PFS, 324 vs 63 days, hazard ratio (HR)=0.36, *p*= 0.0152) and higher objective response rate (ORR, *p*=0.0099). In validation cohort 1 (*n*=377), ns-NSCLC patients with tissue PTPRD mutations had favorable PFS (9.10 vs 4.33 months, HR=0.62, *p*=0.0184) and ORR (*p*=0.013). In validation cohort 2 (*n*=406), ns-NSCLC patients with tissue PTPRD mutations had favorable overall survivals (OS, over 40 vs 11.94 months, HR=0.57, *p*=0.011). In validation cohort 3 (*n*=1137), ns-NSCLC patients with ctDNA PTPRD mutations had longer PFS (6.97 vs 2.73 months, HR=0.63, *p*=0.028) and higher ORR (*p*=0.047). Moreover, it was deleterious mutations in phosphatase domains (phosphatase-mut), rather than other mutations (other-mut), that were responsible of PTPRD’s prediction efficiency. In addition, in validation cohort 3, ctDNA phosphatase-mut also functioned as a predictive biomarker helping identify patients benefiting more from ICIs than chemotherapy (interaction *P* for PFS=0.0506, for OS=0.04). Univariate and multivariate regression analysis revealed that phosphatase-mut was independent on PD-L1 expression and tumor mutation burden (TMB) to predict. In silico analysis based on TCGA LUAD cohort discovered enhanced anti-tumor immunity in phosphatase-mut patients.

**Conclusions:**

Tissue or ctDNA PTPRD phosphatase domain deleterious mutations might function as a both prognostic and predictive biomarker predicting clinical outcomes of ICIs in ns-NSCLC patients, independent on TMB or PD-L1 expression.

**Supplementary Information:**

The online version contains supplementary material available at 10.1186/s12916-021-02075-5.

## Background

Although immune checkpoint inhibitors (ICIs) have recently shown promising survival advantages in treating patients with non-small cell lung cancer (NSCLC) [1, 2], only a few patients respond to current immunotherapy [3]. Therefore, identifying biomarkers that determine responsiveness to ICIs is an imperative goal. To date, programmed death ligand 1 (PD-L1) expression and the tumor mutational burden (TMB) are two predictive biomarkers for ICIs that have been validated prospectively in randomized controlled trials (RCTs) concerning NSCLC. However, both of them have several limitations. Notably, patients with high PD-L1 expression have better prognosis [4], but those with PD-L1 expression <1% could still benefit from ICIs [5]. Additionally, although PD-L1 expression is analogous, some patients still show a non-response to ICIs [6–8]. TMB is a continuous variable, whose optimum cutoff value remains controversial. Various platforms and standards to determine the cutoff value using gene panels are non-uniform [5, 9–11]. When it comes to liquid biopsy, PD-L1 and TMB are more unsatisfactory. Patients with pre-treatment PD-L1+ circulating tumor cells (CTCs) were associated with an inferior prognosis after treatment with PD-(L)1 inhibitors [12–14], in contrast to PD-L1 expression correlating with favorable outcomes in tissue. In the OAK and POPLAR trials [11], bTMB ≥16 was revealed to be a predictive but not prognostic biomarker for ICIs in NSCLC. In a recent study, bTMB ≥6 was a prognostic biomarker for progression-free survival (PFS) in patients treated with ICIs [9], but whether it worked in predicting overall survivals (OS) remains uncertain.

Apart from PD-L1 expression and TMB, recent studies have reported that specific gene alterations, such as KRAS/TP53 [15], STK11/LKB1 [16, 17], EGFR [17], POLD1/POLE [18], TET1 [19], EPHA [20], KEAP1 [21], and NOTCH [22], are linked to responses to ICIs by regulating the tumor immune microenvironment (TIME) and served as biomarkers to predict the outcomes of ICIs. However, they were only prognostic but not predictive, which meant uncapable of helping selecting patients benefit from ICIs. Moreover, it would be more convenient and accessible to more patients if their mutations in ctDNA could also predict, but had not been validated yet.

Receptor-type protein tyrosine phosphatases (PTPRs) are a transmembrane immunoglobulin family comprising more than 20 members, sharing homologous phosphatase domain(s) and distributing along cell membranes [23]. By dephosphorylating target proteins, PTPRs antagonize the activities of protein tyrosine kinases (PTKs) and are involved in signal transduction, regulating tumorigenesis, and the TIME. Taking PTPRD as an example, PTPRD inactivation induced CXCL8 to promote angiogenesis and metastasis in gastric cancer [24]. Loss of PTPRD led to aberrant STAT3 activation and promoted glioma progression [25]. PTPRD deleterious mutations and deletion predicted bevacizumab resistance in metastatic colorectal cancers [26]. The mutational inactivation of PTPRD in glioblastoma was linked to melanoma malignancy [27]. Other PTPRs, including PTPRA [28], PTPRC [29], and PTPRJ [30], are also involved in regulating tumorigenesis and the TIME. More importantly, it was reported that PTPRD interacting with its ligand LRFN4 was related to responses to ICIs in bladder cancers [31] but unclear in NSCLC yet. Hence, we assumed that PTPRs also had assignable functions in response to ICIs in NSCLC.

Here, we report that the tissue and ctDNA PTPRD phosphatase domain mutations functioned as a prognostic biomarker predicting higher objective response rate (ORR) and favorable PFS of ICIs. PTPRD phosphatase domain mutations in tumor tissue predicted OS and that in ctDNA served as a predictive biomarker helping select patients benefiting from ICIs in non-squamous NSCLC (ns-NSCLC). PTPRD prediction effectiveness was independent of TMB/PD-L1 expression and may be attributed to regulating the TIME.

## Methods

### Patients in the discovery cohort

Seventy-three NSCLC patients who received anti-PD-(L)1 antibody therapy were evaluated as the discovery cohort (also named the NCC cohort). These 73 patients were from the National Cancer Center/National Clinical Research Center for Cancer/Cancer Hospital and Chinese Academy of Medical Sciences & Peking Union Medical College and Sun Yat-sen University Cancer Center from December 2016 to December 2018. All 73 patients with NSCLC were part of clinical trials. The inclusion criteria for the qualified patients for this study were as follows: (1) age ≥18 years, (2) Eastern Cooperative Oncology Group (ECOG) performance status of 0–1, (3) advanced or recurrent NSCLC validated by pathology, (4) failure after the first-line platinum-base chemotherapy, and (5) radiologically evaluable according to RECIST v1.1. CT or MRI imaging scans were evaluated by at least 2 qualified investigators independently. The patients’ tumor tissues were sequenced by whole-exon sequencing (WES). This study was approved by the ethics committees of all participating centers (NCC2016JZ-03 and NCC2018-092). All 73 patients provided written informed consent. Clinical characteristics of these 73 patients were listed in Additional file 1: Table S1.

### Public cohorts

Other 7 independent public cohorts were also used in the present study, named the N. Rizvi [32], Hellmann [33], Miao [34], MSKCC-240 [35], NG1661 [10], OAK, and POLAR cohorts [11], respectively. NSCLC patients of NG1661 cohort were separated and named as MSKCC-350 cohort. The data of these 7 public cohorts were collected from corresponding published articles. The details of these 7 public cohorts and the NCC cohort are listed in Additional file 1: Table S2.

Data from the TCGA-LUAD and TCGA-LUSC cohorts were downloaded from cBioPortal [36, 37].

### PTPRD mutations

Non-synonymous mutations, including in-frame/frame-shift deletions/insertions, nonsense, nonstop, splice region, splice site, and missense mutations, were defined as PTPRD mutations in this study. Protein Variation Effect Analyzer (PROVEAN )[38] was applied to predict the influences of the mutations on PTPRD functions. All single-amino acid substitutions and in-frame deletion/insertion/insertion-deletion were analyzed using PROVEAN. The mutations were classified as neutral or deleterious with a cutoff value at −2.5 according to the official instructions. Details about the PROVEAN score/prediction of each mutation are listed in Additional file 1: Table S3. All nonsense, frameshift deletion/insertion, and splice region/site mutations were also defined as deleterious based on their changing whole subsequent amino acid sequences. Deleterious mutations in phosphatase domains were defined as phosphatase-mut (P-mut), and all the other mutations were then defined as other-mut (O-mut).

### Study assessment

In the NCC cohort, the responses were assessed every 6–8 weeks after the first anti-PD-(L)1 treatment until objective disease progression. The objective response rate (ORR) was defined as the percentage of patients with a confirmed complete response (CR) or partial response (PR) by RECIST V.1.1. PFS was defined as the time from the first treatment to the date of progression or death. Patients who had not progressed as of January 1, 2020, were recorded as censored.

In the 7 public cohorts, the responses were assessed according to the description in each published article 195

### Bioinformatics analysis

RNA-seq data from TCGA-LUAD and TCGA-LUSC were used to estimate the infiltrations of immune cells with CIBERSORTx [39] or gene set enrichment analysis (GSEA) with Java GSEA Desktop Application (GSEA v4.0.1) [40]. For GSEA, the HALLMARK and KEGG gene sets were applied, while the other settings were default. To better understand the role of PTPRD mutation in modulating the TIME and the response to ICIs, an immunogenomic pipeline was applied [41]. To explore associations between PTPRD expression and estimation of the infiltrations of immune cells and enriched pathways, only PTPRD-WT samples were adopted.

### Statistical analysis and graphics

Continuous variables were compared by the Mann-Whitney *U* test. The significance of categorical variables (e.g., ORR and PD-L1 status) was evaluated by chi-squared test or Fisher’s exact test. Survival was compared with Kaplan-Meier curves, and the *p* value was calculated by the log-rank test. The hazard ratio (HR) was determined using univariable and multivariable Cox regression. Variables with *p* < 0.1 in the univariable regression were included in the multivariable Cox regression. All the analyses were implemented via GraphPad (v 8.0.3) or R (v 4.0.3). All the graphics were drawn with GraphPad (v 8.0.3) or R (v 4.0.3).

## Results

### Workflow and PTPR mutations in NSCLC patients treated with ICIs

**Fig. 1:**
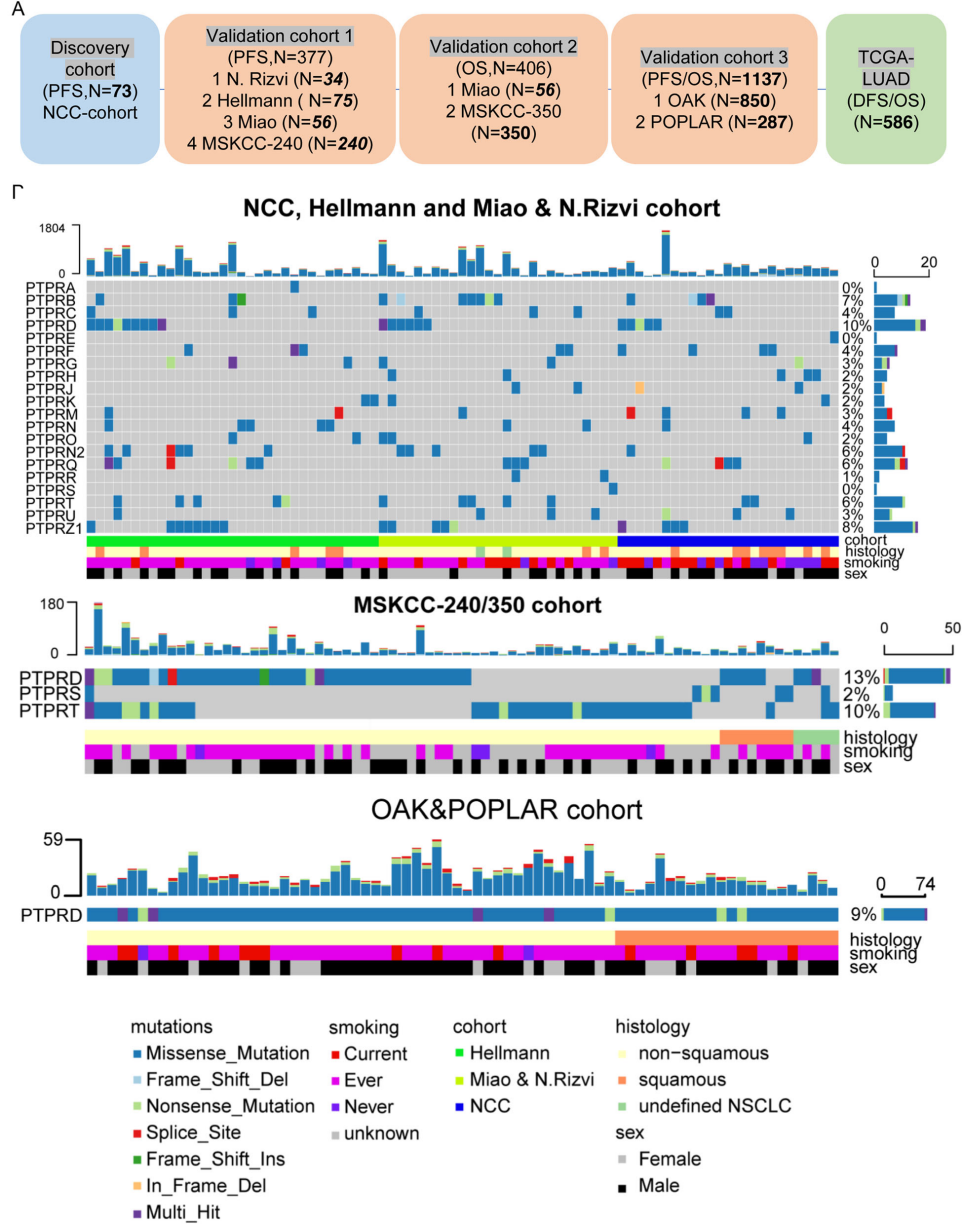
Workflow and PTPR alterations in NSCLC patients treated with ICIs. **a** Workflow of this study. **b** PTPR mutations landscape of multiple cohorts

To investigate the roles of PTPRs in ICI therapies, we utilized a discovery cohort and 3 validation cohorts (Fig. 1a). The discovery cohort included the clinical and WES data of 73 NSCLC patients treated with ICIs. The patient characteristics of the discovery cohort are listed in Additional file 1: Table S1. Validation cohort 1 (*n*=377) consisted of 4 independent cohorts with detailed PFS and objective evaluation of responses, the N. Rizvi (*n*=34), Hellmann (*n*=75), Miao (*n*=56), and MSKCC-240 (*n*=240) cohorts and was used to validate predicting ORR and PFS. Validation cohort 2 (*n*=406) consisted of 2 independent cohorts with detailed OS, the Miao (*n*=56) and MSKCC-350 (*n*=350) cohorts, and was used to validate predicting OS. Validation cohort 3 (*n*=1137) consisted of 2 independent cohorts, the OAK (*n*=850) and POPLAR (*n*=287) cohorts, and was applied to validate the predictive effectiveness in ctDNA. The workflow of this investigation was shown in Fig. 1a.

Among the NCC, Hellmann and Miao and N. Rizvi cohorts (all sequenced by WES), all the PTPRs were mutated, ranging from 0.4% (PTPRA, PTPRE, PTPRS) to 10% (PTPRD) (Fig. 1b upper panel). In MSKCC-240/350 (both were sequenced by the IMPACT panel and shared some common patients), PTPRD, PTPRS, and PTPRD were enrolled in the panels, with mutation rates of 13%, 2%, and 10%, respectively (Fig. 1b middle panel). In the OAK and POPLAR cohorts (both sequenced by Foundation One^TM^ based on ctDNA), only PTPRD was sequenced, with a mutation rate of 9% (Fig. 1b bottom panel).

### Tissue PTPRD mutations was a prognostic biomarker for ICIs in ns-NSCLC

**Fig. 2:**
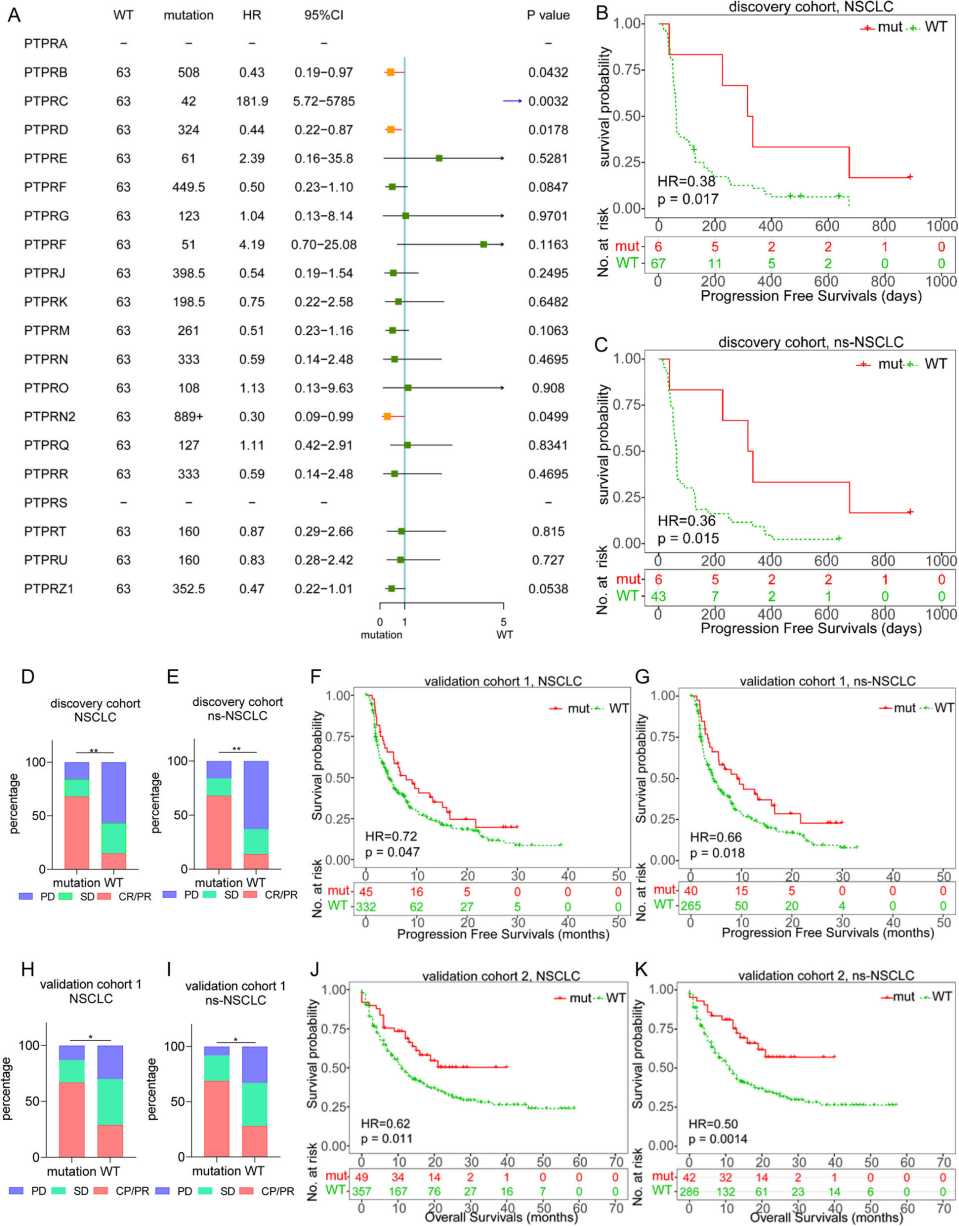
Tissue PTPRD mutation predicts the efficiency of ICIs in non-squamous NSCLC. **a** Forest plot of HR of PTPR mutations for PFS in discovery cohort. **b**, **c** Progression-free survivals of NSCLC (**b**) or ns-NSCLC (**c**) in discovery cohort. **d**, **e** Objective responses of NSCLC (**d**) or ns-NSCLC (**e**) in discovery cohort. **f**, **g** Progression-free survivals of NSCLC (**f**) or ns-NSCLC (**g**) in a validation cohort 1. **h**, **i** ORR of NSCLC (**h**) or ns-NSCLC (**i**) in validation cohort 1. **j**, **k** Overall survivals of NSCLC (**j**) or ns-NSCLC (**k**) in validation cohort 2

We first investigated the hazard ratios (HRs) of PTPR mutations for PFS in NSCLC in the discovery cohort and found that PTPRB (HR=0.43, *p*=0.0432), PTPRD (HR=0.44, *p*=0.0178), and PTPRN2 (HR=0.30, *p*=0.0499) mutations were associated with longer PFS, while PTPRC mutations were linked to a shorter PFS (HR=181.9, *p*=0.0032) (Fig. 2a). With the highest mutation rates among all PTPRs, which meant applicable to more patients, PTPRD was chosen for further investigation. The K-M plotter of PTPRD mutation vs wild type (WT) for PFS (324 vs 63 days, HR=0.38, *p*=0.0169) of NSCLC patients is shown in Fig. 2b. Considering the heterogeneity of responses to ICIs of non-squamous and squamous carcinoma, NSCLC patients were divided into non-squamous or squamous subgroups. In non-squamous NSCLC (ns-NSCLC), PTPRD mutation patients also had a longer median PFS (324 vs 63 days, HR=0.36, *p*=0.0152, Fig. 2c). No PTPRD mutation was found in the squamous subgroup. Among all the NSCLC patients, the objective response rate (ORR) was higher in the PTPRD mutation group than in the WT group (68% vs 15%, *p* <0.01, Fig. 2d), with concordant results in the ns-NSCLC subgroup (68% vs 14%, *p* <0.01, Fig. 2e).

We next validated the associations between PTPRD mutations and PFS/ORR in validation cohort 1. Among all the NSCLC patients, patients with PTPRD mutations showed a longer median PFS (7.95 vs 4.37 months, HR=0.69, *p*=0.047, Fig. 2f), with a similar result in ns-NSCLC (9.10 vs 4.33 months, HR=0.62, *p*=0.0184, Fig. 2g). However, no consistent trend was found in the squamous subgroup (8.38 vs 4.27 months, HR=1.30, *p*=0.72, Additional file 2: Fig. S1a). A favorable ORR was observed in PTPRD mutation patients in both the NSCLC (67% vs 29%, *p*=0.011, Fig. 2h) and ns-NSCLC subgroups (69% vs 28%, *p*=0.013, Fig. 2i).

In validation cohort 2, NSCLC patients with PTPRD mutations had longer median OS (over 40.00 vs 11.00 months, HR=0.57, *p*=0.0112, Fig. 2j). The result was similar in ns-NSCLC (over 40 vs 11.94 months, HR=0.40, *p*=0.0007, Fig. 2k). In the squamous subgroup, an inverse trend was observed (6.00 vs 11.00 months, HR=3.24, *p*= 0.0073, Additional file 2: Fig. S1b).

Details of the PTPRD mutation predicting PFS/OS in the 5 independent cohorts in validation cohorts 1 and 2 are shown in the supplements (Additional file 2: Fig. S1c-d, Fig. S2, Fig. S3).

Collectively, tissue PTPRD mutation predicted a longer PFS/OS and a higher ORR in non-squamous but not squamous lung cancer patients treated with ICIs.

### ctDNA PTPRD mutation is a prognostic biomarker for ICIs in ns-NSCLC

**Fig. 3:**
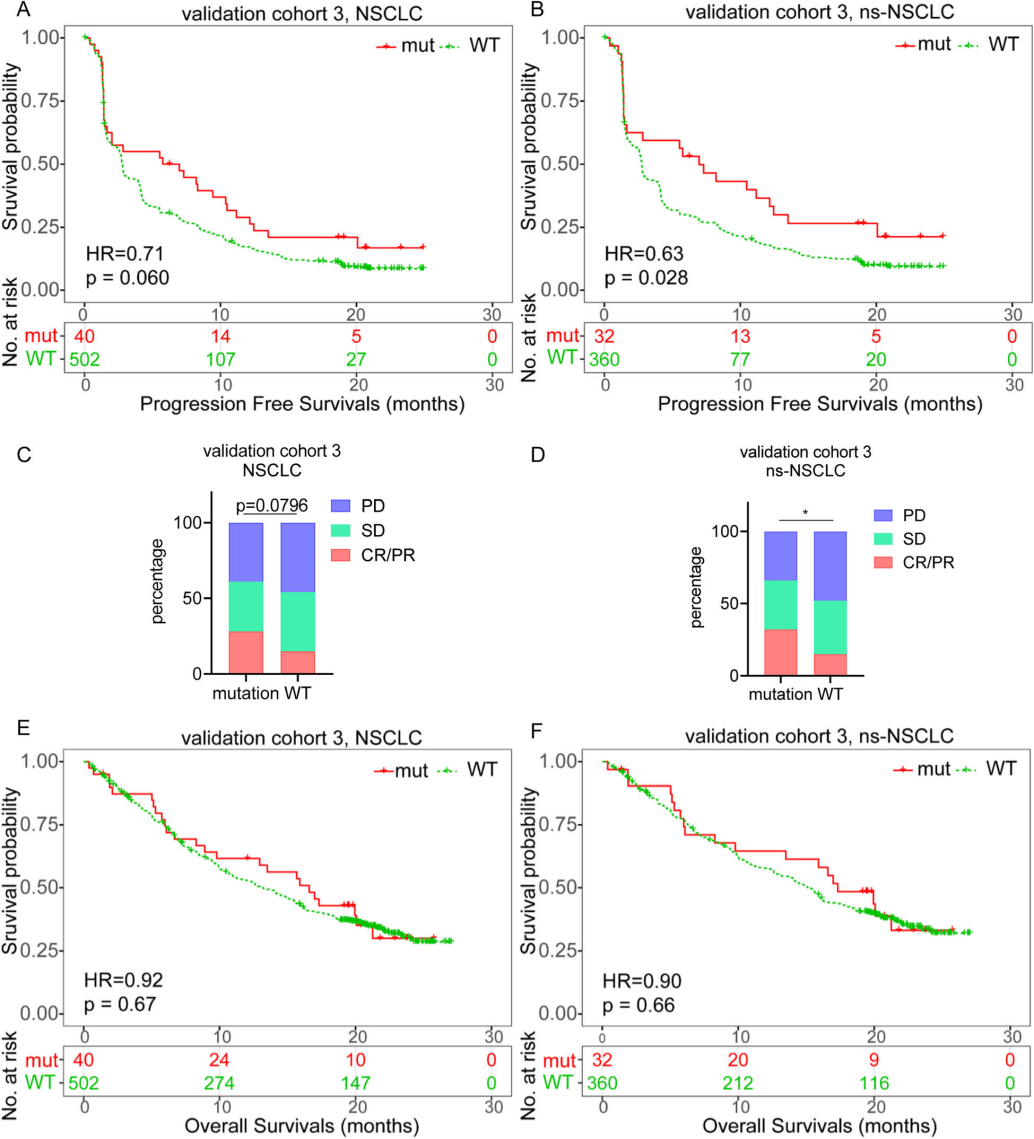
ctDNA PTPRD mutation predict the efficiency of ICIs in ns-NSCLC. **a**, **b** Progression-free survivals of NSCLC (**a**) or ns-NSCLC (**b**) in validation cohort 3. **c**, **d** Objective responses of NSCLC (**c**) or ns-NSCLC (**d**) in different groups. **e**, **f** Overall survivals of NSCLC (**e**) or ns-NSCLC (**f**) in validation cohort 3

In validation cohort 3, which was applied to test whether the ctDNA PTPRD mutations’ prediction efficiency was consistent with tissue PTPRD mutations, there was a tendency that median PFS of the ctDNA PTPRD mutations was longer than that of the WT group in NSCLC, despite not statistically significant (6.36 vs 2.36 months, HR=0.71, *p*=0.0603, Fig. 3a). In ns-NSCLC, the median PFS of the ctDNA PTPRD mutations was longer than that in the WT group (6.97 vs 2.73 months, HR=0.63, *p*=0.0282, Fig. 3b). Details of these 2 independent cohorts are shown in Additional file 2: Fig. S4a-d. In the squamous subgroup, the median PFS was not different between the PTPRD mutation and WT groups (2.00 vs 2.86 months, HR=1.28, *p*=0.49, Additional file 2: Fig. S1e). Details of these 2 independent cohorts are shown in Additional file 2: Fig. S1f-g. Additionally, for NSCLC patients, ORR in the mutation group was not different from that in the WT group (28% vs 15%, *p*=0.0796, Fig. 3c). While in ns-NSCLC, ORR in the mutation group was higher than that in the WT group (32% vs 15%, *p*=0.047, Fig. 3d).

The median OS difference between PTPRD mutation and wild type was not significant in the NSCLC (16.6 vs 13.2 months, HR=0.92, *p*=0.670, Fig. 3e), ns-NSCLC (17.3 vs 15.2 months, HR=0.90, *p*=0.662, Fig. 3f) or squamous subgroup (8.90 vs 8.48 months, HR=1.29, *p*=0.619, Additional file 2: Fig. S1h). Similar results were observed in 2 individual cohorts (Additional file 2: Fig. S4e-h, Fig. S1i-j).

Taken together, ctDNA PTPRD mutation could also predict longer PFS and higher ORR but not OS in ns-NSCLC patients treated with ICIs.

### Phosphatase-mut of PTPRD is responsible for function as a prognostic biomarker

**Fig. 4:**
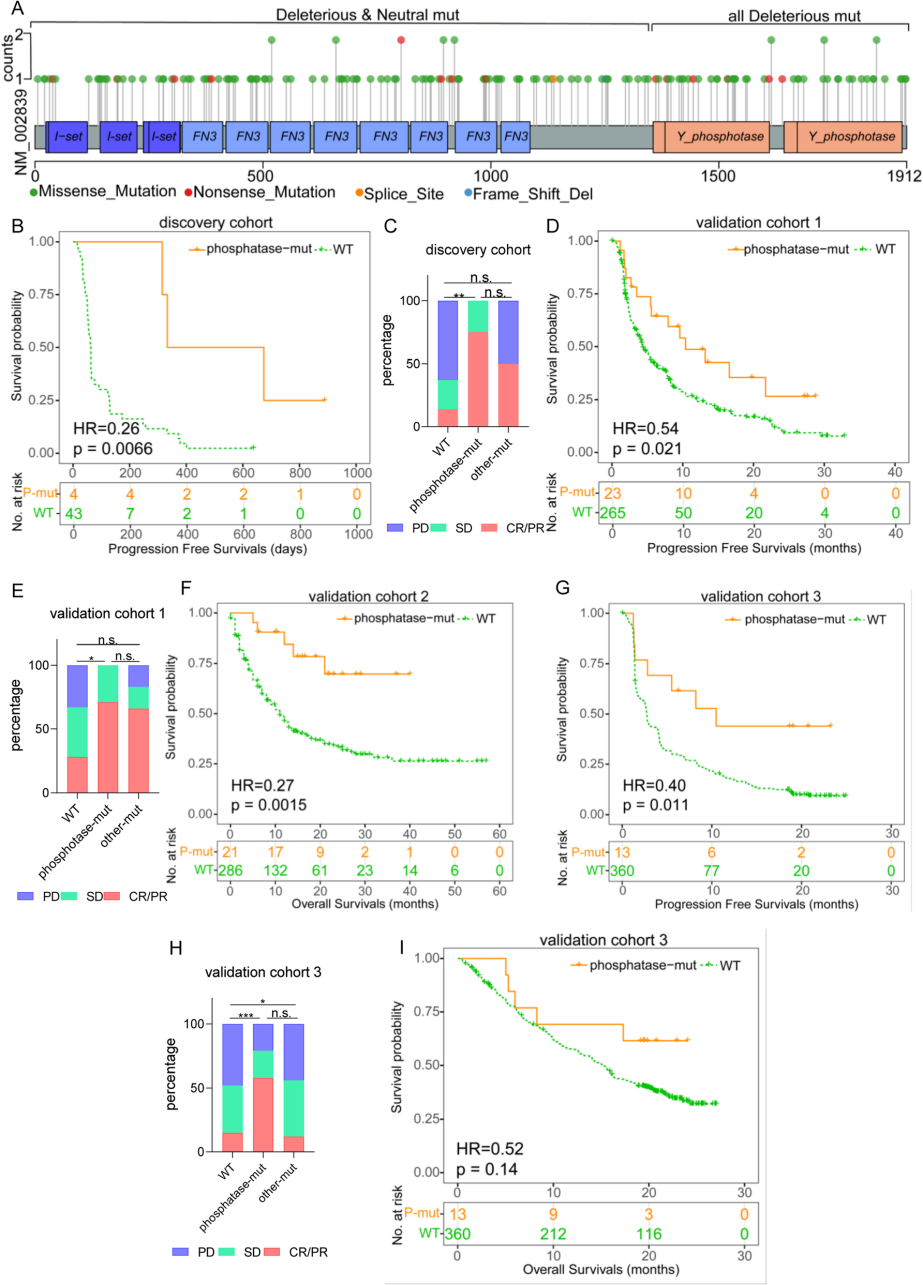
Phosphatase-mut of PTPRD was responsible of function as a prognostic biomarker. **a** Distribution of PTPRD mutations and overlap of mutation types (deleterious/neutral) and sites (phosphatase/other domains). **b**, **c** PFS (**b**) and ORR (**c**) of different groups in discovery cohort. **d**, **e** PFS (**d**) and ORR (**e**) of different groups in validation cohort 1. **f** OS of different groups in validation cohort 2. **g**, **i** PFS (**g**), ORR (**h**), and OS (**i**) of different groups in validation cohort 3

We subsequently summarized the distributions of the mutation sites of PTPRD, finding that the mutation sites were diffuse with no obvious hotspot mutation (Fig. 4a). To evaluate the influence of specific single mutation on PTPRD biological functions, we applied the online tool PROVEAN to predict the influences of each mutation (see the “Methods” section). Considering that different domains of PTPRD have individual functions and that PTPRD mainly functions as a phosphatase through its phosphatase domains, we defined two mutation groups as phosphatase-mut and other-mut comprehensively based on neutral or deleterious and locations (phosphatase or other domains). Deleterious mutations in phosphatase domains were defined as phosphatase-mut (P-mut). The other mutations were defined as other-mut (O-mut). Interestingly, there were no neutral mutations in phosphatase domains (Fig. 4a). We next analyzed the differences in the predictive efficiency of these two mutation groups.

In ns-NSCLC of the discovery cohort, phosphatase-mut had a longer median PFS than WT (503.5 vs 63.00 days, HR=0.26, *p*=0.0066, Fig. 4b) or other-mut (503.5 vs 132 days, HR=0.18, *p*= 0.0177, see Additional file 2: Fig. S5a). The median PFS of other-mut patients was not significantly different from that of WT patients (132 vs 63 days, HR=1.01, *p*= 0.99, Additional file 2: Fig. S5b). The ORR of phosphatase-mut was higher than that of WT (75% vs 14%, *p*<0.01, Fig. 4c). The differences in ORR between other-mut and WT or phosphatase-mut were not significant (both *p* ≥0.05, Fig. 4c).

In ns-NSCLC of validation cohort-1, the median PFS of phosphatase-mut was longer than that of WT (10.39 vs 4.33 months, HR=0.54, *p*= 0.0213, Fig. 4d). The difference between other-mut and phosphatase-mut (HR=0.69, *p*=0.3288, Additional file 2: Fig. S5c) or WT was not significant (HR=0.75, *p*=0.3267, Additional file 2: Fig. S5d). The ORR of phosphatase-mut was higher than that of WT (71% vs 28%, *p*=0.035, Fig. 4e). The difference in ORR between other-mut and WT or phosphatase-mut was not significant (both *p* ≥0.05, Fig. 4e).

In ns-NSCLC of validation cohort 2, the median OS of phosphatase-mut was longer than that of WT (over 40.00 vs 11.94 months, HR=0.27, *p*=0.0015, Fig. 4f). The difference between phosphatase-mut and other-mut (over 40 vs 19.00 months, HR=0.40, *p*=0.0818, Additional file 2: Fig. S5e) or other-mut and WT was not significant (19.00 vs 11.94 months, HR=0.65, *p*=0.1769, Additional file 2: Fig. S5f).

In ns-NSCLC of validation cohort 3, the median PFS of phosphatase-mut was longer than that of WT (10.78 vs 2.73 months, HR=0.40, *p*= 0.0106, Fig. 4g). The difference between phosphatase-mut and other-mut (10.48 vs 5.75 months, HR=0.51, *p*=0.1175, Additional file 2: Fig. S5g) or other-mut and WT was not significant (5.75 vs 2.73 months, HR=0.85, *p*=0.4988, Additional file 2: Fig. S5h). The ORR of phosphatase-mut was higher than that of WT (58% vs 15%, *p*=0.0002, Fig. 4h). Compared with other-mut, the ORR of WT was higher (15% vs 12%, *p*=0.021, Fig. 4h). The difference between other-mut and phosphatase-mut was not significant (58% vs 12%, *p*=0.804, Fig. 4h). The median OS between phosphatase-mut and WT was not significant (over 24.09 vs 15.18 months, HR=0.52, *p*=0.1422, Fig. 4i). The difference between phosphatase-mut and other-mut (over 24.09 vs 16.59 months, HR=0.46, *p*=0.1225, Additional file 2: Fig. S5i) or other-mutation and WT was not significant (16.59 vs 15.18 months, HR=0.82, *p*=0.4825, Additional file 2: Fig. S5j).

The details of PTPRD phosphatase-mut predicting the PFS/OS of ICIs in each tissue or ctDNA cohort are shown in Additional file 2: Fig. S6 (tissue cohorts) and Fig. S7 (ctDNA cohorts).

These data suggest that PTPRD phosphatase-mut can predict the PFS, OS, and ORR of ICIs, while other-mut cannot.

### ctDNA PTPRD phosphatase-mut is a predictive biomarker for ICIs in ns-NSCLC

**Fig. 5:**
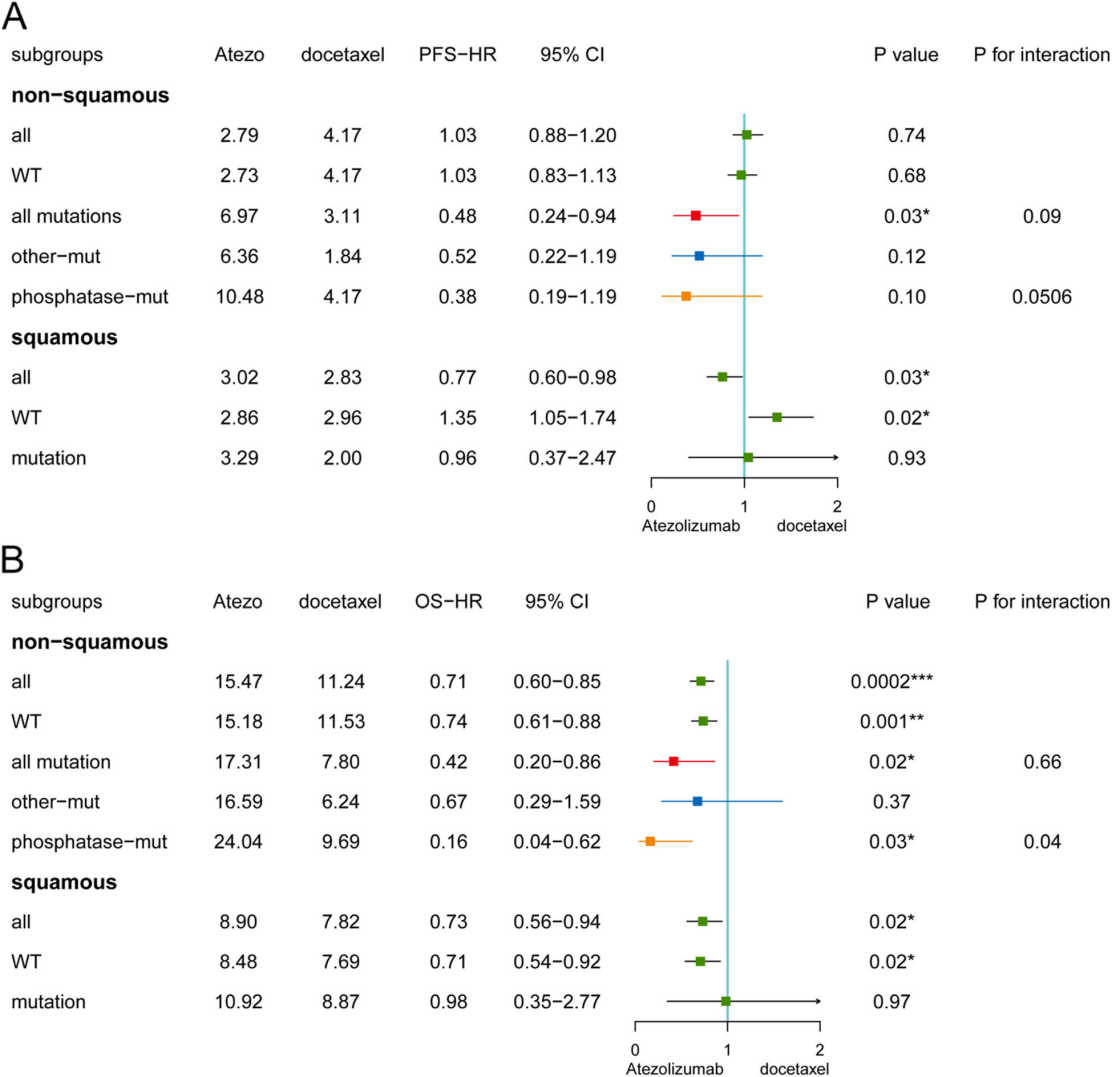
ctDNA PTPRD phosphatase-mut was a predictive biomarker for ICIs in ns-NSCLC. **a**, **b** PFS (**a**) and OS (**b**) HR forest plot of Atezolizumab in different subgroups in validation cohort 3. *p<0.05; **p<0.01; ***p<0.001; ****p<0.0001

To investigate whether PTPRD phosphatase-mut was a predictive biomarker for selecting patients who could benefit more from ICIs (Atezolizumab) than chemotherapy (docetaxel), we firstly studied whether PTPRD phosphatase-mut was a predictive biomarker for PFS in several subgroups (Fig. 5a). PTPRD WT was not a predictive biomarker for Atezolizumab (*p* ≥0.05, Fig. 5a). In the mutation subgroup, the median PFS of Atezolizumab and docetaxel was 6.97 vs 3.11 months (HR=0.48, *p*=0.03, *P* value for interaction=0.09); in the other-mut subgroup, the median PFS of Atezolizumab and docetaxel was 6.36 vs 1.84 months (HR=0.52, *p*=0.12); in the phosphatase-mut subgroup, the median PFS of Atezolizumab and docetaxel was 10.48 vs 4.17 months (HR= 0.38, *p*=0.10, *p* value for interaction=0.0506). Additionally, PTPRD mutations were not a biomarker for ICIs in the squamous subgroup (median PFS: 3.29 vs 2.00 months, HR=0.96, *p*=0.93).

We further investigated the roles of PTPRD in predicting OS between Atezolizumab and docetaxel (Fig. 5b). In the ns-NSCLC group (median OS: 15.47 vs 11.24 months, HR=0.71, *p*=0.0002), WT group (median OS: 15.18 vs 11.53 months, HR=0.74, *p*=0.001), all-mutation group (median OS: 17.31 vs 7.8 months, HR=0.42, *p*= 0.0176), and phosphatase-mut subgroup (median OS: 24.04 vs 9.69 months, HR=0.16, *p*=0.0273), but not in the other-mut subgroup (median OS: 16.59 vs 6.24 months, HR=0.57, *p*=0.368), Atezolizumab had a longer median OS than docetaxel (Fig. 5b). The *p* values for interaction between WT/all mutations or WT/phosphatase-mut and Atezolizumab/docetaxel were 0.66 and 0.04, respectively. In the mutation subgroup of squamous patients, no difference was found between the OS of Atezolizumab and docetaxel (median OS: 10.92 vs 8.87 months, HR=0.98, *p*=0.97).

Taken together, our findings revealed that PTPRD phosphatase-mut is a predictive biomarker that can predict the PFS/OS between patients treated with Atezolizumab or docetaxel.

### PTPRD’s prediction effectiveness was independent on TMB or PD-L1 expression

**Fig. 6:**
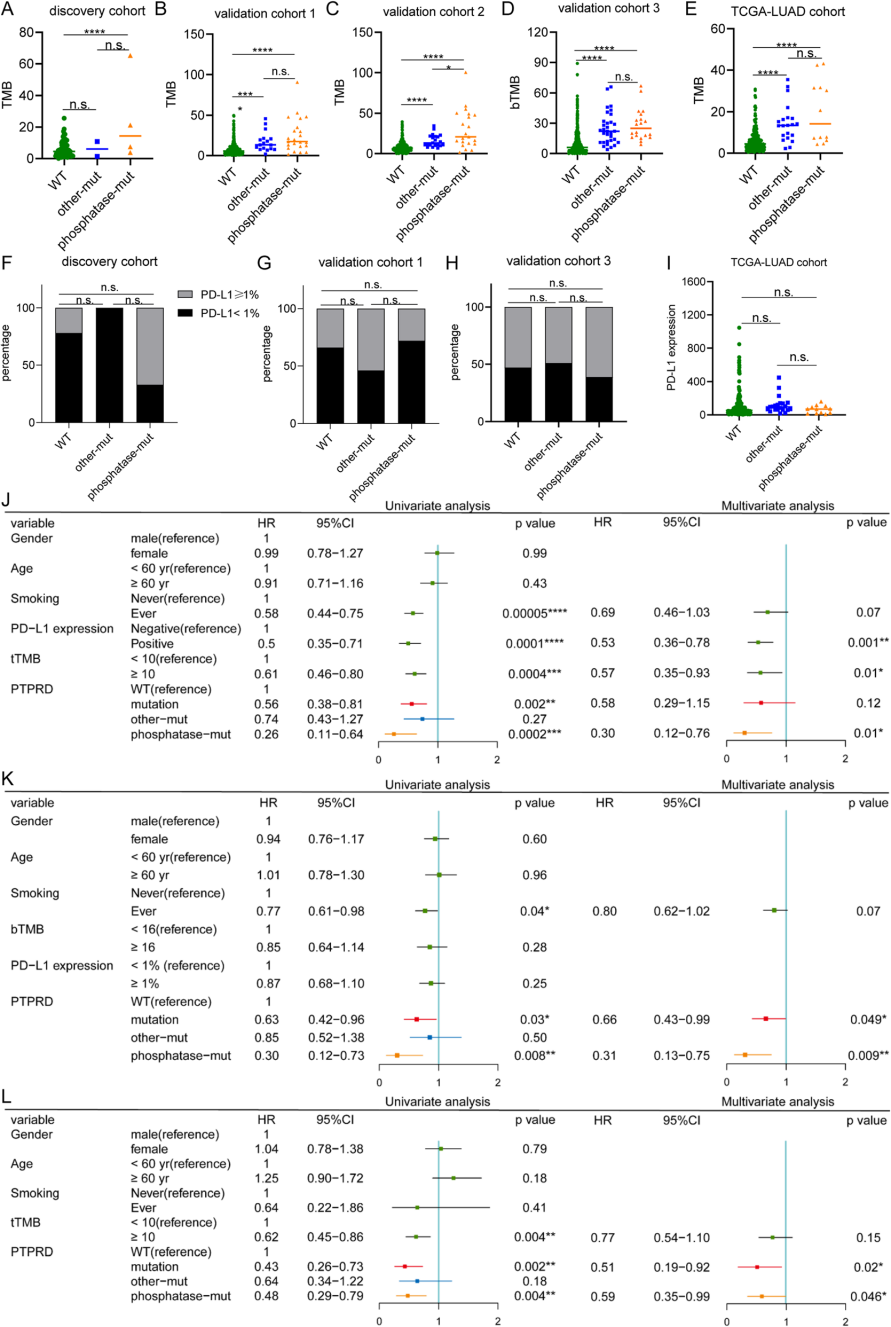
Associations between PTPRD mutation and TMB, PD-L1 expression. **a**, **e** TMB of WT, other-mut, phosphatase-mut group in discovery cohort (**a**), validation cohort 1 (**b**), validation cohort 2 (**c**), validation cohort 3 (**d**), and TCGA-LUAD cohort (**e**). **f**, **i** PD-L1 expression of WT, other-mut, and phosphatase-mut group in discovery cohort (**f**), validation cohort 1 (**g**), validation cohort 3 (**h**), and TCGA-LUAD cohort (**i**). **j**–**l** Forest plots of univariate and multivariate cox regression analysis of merged cohort of discovery and validation cohort 1 (**j**), validation cohort 3 (**k**), and validation cohort 2 (**l**). *p<0.05; **p<0.01; ***p<0.001; ****p<0.0001; n.s., no significance

Considering TMB and PD-L1 expression were two widely applied biomarkers for ICIs, we tested if PTPRD predicting was dependent on them. We calculated and compared the difference in TMB and PD-L1 expression among PTPRD WT, other-mut, and phosphatase-mut groups. In the discovery cohort, the TMB of phosphatase-mut was higher than that of WT (24.48 vs 6.18, *p* <0.0001, Fig. 6a). No difference was found between WT and other-mut or phosphatase-mut and other-mut (both *p* ≥0.05, Fig. 6a). In validation cohort 1, the TMB of WT was lower than that of phosphatase-mut (7.93 vs 24.90, *p* <0.0001, Fig. 6b) and other-mut (7.93 vs 17.11, *p* <0.0001, Fig. 6b), while no difference was found between other-mut and phosphatase-mut (17.11 vs 24.90, *p*=0.179, Fig. 6b). In validation cohort 2, the TMB of phosphatase-mut was higher than that of other-mut (30.13 vs 16.00, *p*=0.015, Fig. 6c) and WT (30.13 vs 7.597, *p* <0.0001, Fig. 6c). The TMB of other-mut was also higher than that of WT (16.00 vs 7.597, *p* <0.0001, Fig. 6c). In validation cohort 3, the TMB of WT was lower than that of other-mut (9.76 vs 24.8, *p* <0.0001, Fig. 6d) and phosphatase-mut (9.76 vs 28.7, *p* <0.0001, Fig. 6d). The TMB of other-mut and phosphatase-mut was not different (24.8 vs 28.7, *p* =0.40, Fig. 6d). In the TCGA-LUAD cohort, the TMB of WT was lower than that of other-mut (6.24 vs 11.49, *p* <0.0001, Fig. 6e) and phosphatase-mut (6.24 vs 19.79, *p* <0.0001, Fig. 6e). The TMB of other-mut and phosphatase-mut was not different (11.49 vs 19.79, *p* =0.218, Fig. 6e).

We also investigated if there was any association between PTPRD mutation and PD-L1 expression. In the discovery cohort, validation cohort 1 and validation cohort 3, no difference was found in the PD-L1 expression among the PTPRD WT, other-mut, and phosphatase-mut groups (all *p*≥0.05, Fig. 6f–h). In the TCGA-LUAD cohort, PD-L1 mRNA expression was similar in the PTPRD WT, other-mut, and phosphatase-mut groups (all *p*≥0.05, Fig. 6i).

Considering the difference in the TMB between WT and phosphatase-mut, we discussed whether phosphatase-mut was an independent biomarker in univariate and multivariate Cox regression analyses of these cohorts. Due to the limited number of cases in the discovery cohort (49 cases of ns-NSCLC), it was combined with validation cohort 1 to enhance the testing efficiency. In this merged cohort, PTPRD phosphatase-mut (HR=0.26 (*p*=0.0002) in univariate analysis; HR=0.30 (*p*=0.01) in multivariate analysis, Fig. 6j), but not other-mut (HR=0.74, *p*=0.27 in univariate analysis, Fig. 6j), was an independent biomarker to predict the PFS of ICIs. In validation cohort 3, PTPRD phosphatase-mut was an independent biomarker to predict the PFS of ICIs (HR=0.30 (*p*=0.008) in univariate analysis; HR=0.31 (*p*=0.009) in multivariate analysis, Fig. 6k), while other-mut was not (HR=0.85, *p*=0.50 in univariate analysis, Fig. 6k). In validation cohort 2, phosphatase-mut was also a dependent biomarker to predict the OS of ICIs (HR=0.48 (*p*=0.004) in univariate analysis; HR=0.59 (*p*=0.046) in multivariate analysis, Fig. 6l), while other-mut was not (HR=0.64, *p*=0.18 in univariate analysis, Fig. 6l).

Our data suggest that despite associations with TMB, PTPRD phosphatase-mut was a biomarker for predicting the efficiency of ICIs independent on TMB or PD-L1 expression.

### PTPRD phosphatase-mut predictive efficiency is independent on TP53/EGFR/KRAS mutation

To verify whether the PTPRD phosphatase-mut predictive efficiency was independent on other genes mutations, which were reported fundamental in response to ICIs, including TP53, EGFR, KRAS, STK11, and KEAP1, we tested whether PTPRD was co-mutated or exclusively mutated with such genes. In the NGS cohorts (Hellmann, Miao, N.Rizvi, and NCC cohort), there was no neither co-mutation nor exclusive mutation between PTPRD and these genes (all *p*≥0.05, Additional file 2: Fig. S8a). In MSKCC-240 and MSKCC-350 cohort, which were sequenced by IMPACT panel, PTPRD was co-mutated with TP53 (*p*<0.05), but not with other genes (all *p*≥0.05, Additional file 2: Fig. S8b). In OAK/POPLAR cohort, which was sequenced by Foundation One panel, PTPRD was co-mutated with TP53 (*p*<0.05), but not with other genes (all *p*≥0.05, Additional file 2: Fig. S8c). In TCGA-LUAD cohort, which was sequenced by WES, there was no neither co-mutation nor exclusive mutation between PTPRD and these genes (Additional file 2: Fig. S8d).

As TP53 was found to be co-mutated with PTPRD, and EGFR, KRAS were two common mutations in lung adenocarcinoma and found to have great influences on responses to ICIs, we calculated PTPRD phosphatase-mut predictive efficiency in the TP53/EGFR/KRAS mutation/WT subgroups in different cohorts. In discovery cohort, no PTPRD phosphatase-mut was identified in the TP53 WT or EGFR mutation subgroup. In the WT KRAS subgroup (HR=0.21, *p*=0.0078, Additional file 2: Fig. S8e), but not the mutant KRAS subgroup (HR=0.72, *p*=0.75, Additional file 2: Fig. S8e), phosphatase-mut was a biomarker for PFS, but the *p* value for interaction was 0.25 (Additional file 2: Fig. S8e). Regarding validation cohort 1, in the EGFR-WT and KRAS-WT subgroups, but not in the other subgroups, PTPRD phosphatase-mut was a biomarker for PFS (HR=0.52 (*p*=0.04) and HR=0.47 (*p*=0.01), respectively, Additional file 2: Fig. S8f). While the *p* values for interaction of each gene were 0.5, 0.91, and 0.15 (Additional file 2: Fig. S8f). Regarding validation cohort 2, in the TP53-mut, EGFR-WT, and KRAS-WT subgroups, but not in the other subgroups, PTPRD phosphatase-mut was a biomarker for OS (HR=0.42 (*p*=0.0034), HR=0.48 (*p*=0.01), and HR=0.40 (*p*=0.0063), respectively, Additional file 2: Fig. S8g). While the *p* values for interaction of each gene were 0.79, 0.99, and 0.28, respectively (Additional file 2: Fig. S8g). In validation cohort 3, no PTPRD phosphatase-mut was identified in the EGFR-mut and KRAS-mut subgroups. PTPRD phosphatase-mut was a biomarker for PFS (HR=0.31 (*p*=0.04), HR=0.30 (*p*=0.03), HR=0.31 (*p*=0.0049), HR=0.30 (*p*=0.0046), respectively, Additional file 2: Fig. S8h) but not OS (Additional file 2: Fig. S8i) in both the TP53-WT and mutation subgroups. The *p* value for the interaction of PTPRD phosphatase-mut and TP53 mutation/WT was 0.914 for PFS-HR (Additional file 2: Fig. S8h) and 0.515 for OS-HR (Additional file 2: Fig. S8i).

Taken together, although PTPRD was co-mutated with TP53 in MSKCC-240, MSKCC-359 and OAK/POPLAR cohort, PTPRD phosphatase-mut predictive efficiency was independent of TP53/EGFR/KRAS mutation.

### Bioinformatic analysis revealing potential mechanism by which PTPRD regulated the tumor immune microenvironment

**Fig. 7:**
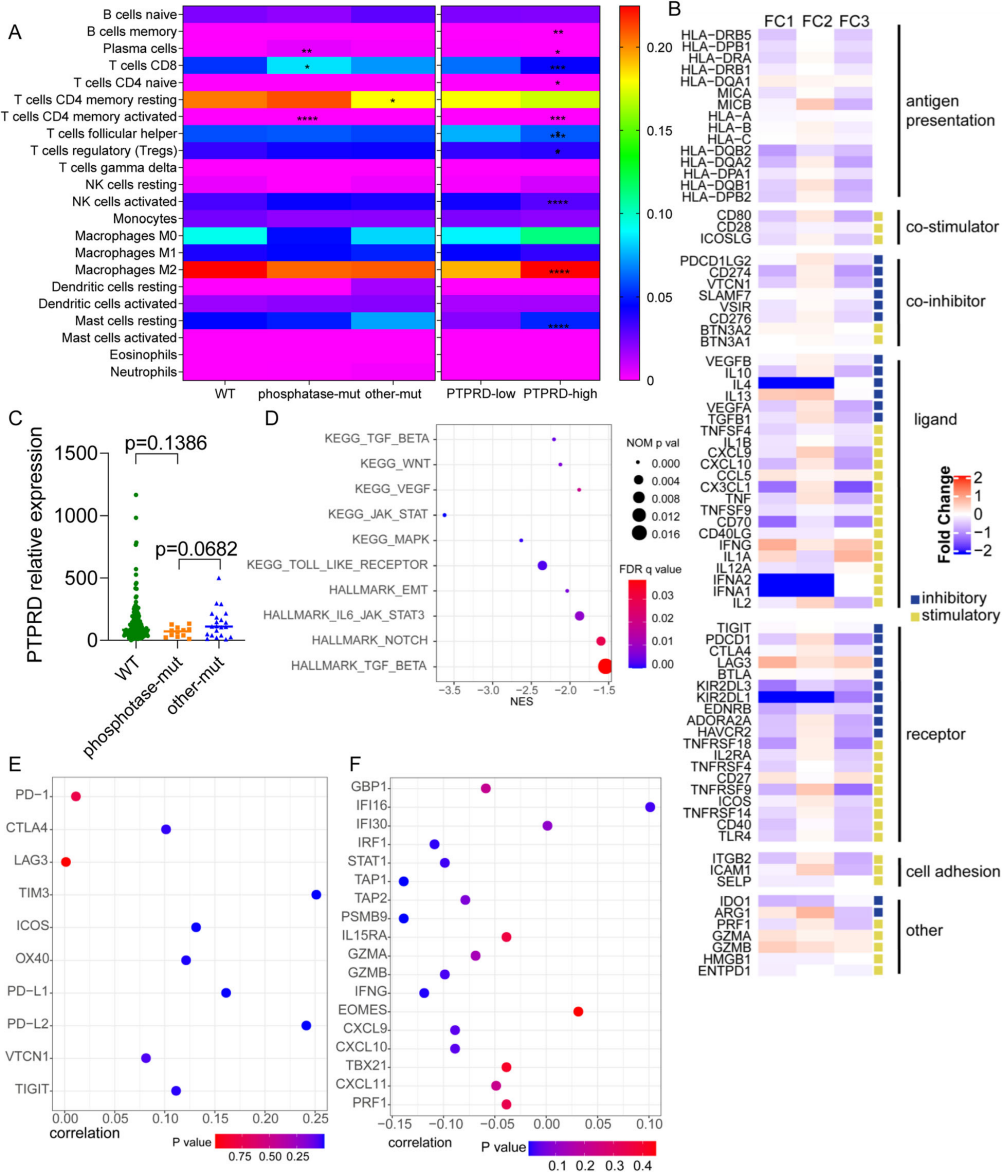
Mechanisms of PTPRD regulating tumor immune microenvironment. **a** Estimation of 22 immune cell infiltrations of PTPRD WT, phosphatase-mut, and other-mut (left panel) or PTPRD low and high (right panel). **b** Heatmap depicting the log2-fold change in the expression level of immune-related genes across PTPRD mutation types (FC1: other-mut/WT, FC2: phosphatase-mut/WT, FC3: phosphatase-mut/other-mut). Blue indicated downregulation and red indicated upregulation. **c** PTPRD expressions across PTPRD mutations in TCGA-LUAD. **d** GSEA enrichments in comparisons between PTPRD low and high. **e**, **f** Correlation between PTPRD and genes among immune checkpoint signature (**e**) and T effector and IFN-γ gene signature (**f**). *p<0.05; **p<0.01; ***p<0.001; ****p<0.0001

The tumor immune microenvironment (TIME) is fundamental to the ICI anti-tumor efficiency. To reveal the potential biological process underlying PTPRD prediction, we focused on whether and how PTPRD regulated the TIME. First, we estimated the infiltration of 22 types of immune cells with CIBERSORTx in the TCGA-LUAD cohort, including CD8+ T cells, regulatory T cells (Tregs), and type 2 macrophages. Compared with WT, PTPRD phosphatase-mut samples had more plasma cells and memory-activated CD4 T cells and CD8+ T cells (all *p*<0.05, Fig. 7a, left panel). Other-mut samples had fewer memory resting CD4+ T cells than WT (*p*<0.05, Fig. 7a, left panel). No difference was found in immune cell infiltration between phosphatase-mut and other-mut samples (all *p*≥0.05, Fig. 7a, left panel). We also estimated the infiltration of 22 types of immune cells by PTPRD expression. The PTPRD-high group (≥median expression) had fewer plasma cells, CD8+ T cells, memory-activated CD4+ T cells, follicular helper T cells, and Tregs, and more activated NK cells, memory B cells, and M2 macrophages, than the PTPRD-low group (<median expression) (all *p*<0.05, Fig. 7a right panel).

Second, to discover how PTPRD mutation regulating vital immune molecules, an immunogenomic pipeline was applie d[41]. Fold changes of PTPRD WT, phosphatase-mut, and other-mut were shown in the heatmap (Fig. 7b, FC1: other-mut/WT, FC2: phosphatase-mut/WT, FC3: phosphatase-mut/other-mut). Compared with WT or other-mut, phosphatase-mut samples had more expression of HLA, co-stimulators, stimulatory ligand and receptor, cell adhesion, and other molecules, despite several co-inhibitors were also elevated.

We failed to find different enriched pathways by Gene Set Enrichment Analysis (GSEA) or different expression patterns of the immune checkpoint signature and T-effector and IFN-γ gene signature among PTPRD WT/phosphatase-mut/other-mut subgroups in the TCGA-LUAD cohort. This reason may be attributed to the limited mutation cases in TCGA-LUAD (12 phosphatase-mut cases and 21 other-mut cases).

Considering that there was a tendency for PTPRD phosphatase-mut to have lower mRNA expression (Fig. 7c), which was also important for biological PTPRD functions, we attempted to reveal the enriched pathways of PTPRD expression and correlations between PTPRD expression and the immune checkpoint signature and T-effector and IFN-γ gene signature. GSEA revealed that TGF-β signaling, WNT signaling, VEGF signaling, JAK_STAT signaling, MAPK signaling, and TOLL-like-receptor signaling in the KEGG library and epithelial-mesenchymal-transition signaling, IL-6_JAK signaling, NOTCH signaling, and TGF-β signaling in the HALLMARK library were enriched in the PTPRD-high group (all NES≤1.5, normalized *p*<0.05, FDR *q*<0.05, Fig. 7d). PTPRD correlated with several genes in the immune checkpoint signature, including CTLA4, TIM3, ICOS, OX40, PD-L1, PD-L2, VTCN1, and TIGIT (all *R*>0, *p*<0.05, Fig. 7e), and negatively correlated with some genes in the T-effector and IFN-γ gene signature, including IFI16, IRF1, STAT1, PSMB9, GZMB, IFN-γ, CXCL9, and CXCL10 (all *R*<0, *p*<0.05, Fig. 7f).

The above data suggest that the phosphatase-mut group had more anti-tumor immune cells and exhibited with a “hot” TIME. Higher PTPRD expression was associated with fewer anti-tumor, more pro-tumor immune cells. Additionally, PTPRD correlated positively with the immune checkpoint signature and negatively with the T-effector and IFN-γ gene signature.

GSEA was also applied to analyze the potential downstream pathways regulated by PTPRD in TCGA-LUSC cohort. The enriched pathway spectrum, including UV response and Hedgehog signaling pathway, was totally different with that in TCGA-LUAD cohort. Those pathways that were reported to be closely related to response to ICIs, including TGF-β signaling, IFN-γ signaling, and WNT signaling, were all not enriched in PTPRD-high group in TCGA-LUSC cohort (Additional file 1: Table S4). The difference of enriched pathways may help explain why PTPRD mutations could not serve as a biomarker for ICIs in squamous lung cancers.

### PTPRD is not a prognostic biomarker of ns-NSCLC treated with surgery and adjuvant chemotherapy

Our data showed that PTPRD phosphatase-mut was not a prognostic biomarker for chemotherapy (*p* for interaction<0.05, Fig. 5a, b). We subsequently analyzed the disease-free survival (DFS)/OS of patients who underwent surgery plus (neo)adjuvant chemotherapy in the TCGA-LUAD cohort. Neither PTPRD mutation nor phosphatase-mut nor PTPRD mRNA expression could predict DFS (all *p*≥0.05, Additional file 2: Fig. S9a-c). Similar results were observed for OS (all *p*≥0.05, Additional file 2: Fig. S9d-f).

Taken together, PTPRD mutation/expression was not a prognostic biomarker of non-squamous lung cancer for chemotherapy and surgery.

### Predicting prognosis efficiency of PTPRD mutation in other cancer types

Base on a public cohort, we analyzed the predictive efficiency of PTPRD mutation in other cancer types, including colorectal cancer, bladder cancer, head and neck cancer, esophagogastric cancer, melanoma, and unknown primary cancers. For melanoma patients, PTPRD mutation group had longer median OS than WT (over 80.00 vs 41.00 months, HR=0.55, *p*=0.0215, Additional file 2: Fig. S10a). For the bladder cancer patients, median PFS of PTPRD mutations group was not different from WT (over 33.00 vs 16.00 months, HR=0.29, *p*=0.062, Additional file 2: Fig. S10b). For other patients (esophagogastric cancer, bladder cancer, head and neck cancer, colorectal cancer, and unknown primary), the median PFS of PTPRD mutation group was not different from WT (all *p* ≥0.05, Additional file 2: Fig. S10c-f).

In general, PTPRD mutations may also predict OS for melanoma patients with ICIs but still need more validation.

## Discussion

To the best of our knowledge, an ideal biomarker for ICIs should be both prognostic and predictive, which means being capable of both predicting the outcomes (including ORR, PFS, and OS) of ICIs and helping select patients benefiting more from ICIs than other therapies [42]. PD-L1 expression and TMB were not satisfactory enough at present. Patients with PD-L1 expression <1% could also benefit from ICIs [5], bTMB≥16 was predictive but not prognostic [11]. Additionally, both PD-L1 expression and TMB were continuous variables without clearly defined cutoff points [43] and varied largely among different detection platforms and method s[44–46]. Hence, in addition to PD-L1 and TMB, other biomarkers are urgently needed.

In contrast to PD-L1 expression and TMB, specific gene mutations are easily detected using the next-generation sequencing assays and easily classified as mutation and wild type. These biomarkers, such as TP53/KRAS [15, 17], STK11 [16], POLE/POLD1 [18], and EHPA [20] mutations, were promising but they were not both prognostic and predictive. Additionally, some of them were not validated in ctDNA, which overcame errors caused by tumor heterogeneity during tissue biopsy and was non-invasive and easy to track dynamically. Biomarkers both prognostic and predictive and applicable to tissue and ctDNA are expected.

Here, we discovered that PTPRD phosphatase-mut could predict the outcomes in terms of ORR and PFS in discovery cohort and validate in 3 consolidated cohorts (total *n*=1920) that tissue or ctDNA PTPRD phosphatase-mut could predict ORR, PFS, and OS of ICIs, in non-squamous NSCLC, although it failed to predict OS in validation cohort 3. It could be explained by dual associations between ctDNA PTPRD mutations and OS. Our recent study reported that although higher bTMB correlated with a higher ORR and longer PFS, while it also correlated with a higher maximum somatic allele frequency, indicating a higher tumor burden and thus linked to shorter OS [47]. Hence, the final association between bTMB and OS was not significant. In our present study, detection of PTPRD phosphatase-mut took enough ctDNA as a prerequisite, which was based on higher tumor burden. Consequently, in this case, ctDNA PTPRD mutations were associated with poor OS. On the other hand, when treated with ICIs, patients with ctDNA PTPRD mutation could benefit from ICIs and thus had better OS. Taken together, ctDNA PTPRD mutations’ entire influence on OS was dual and of not statistical significance. In addition, PTPRD phosphatase-mut was also a predictive biomarker selecting patients who could benefit more from ICIs than chemotherapy. It is importantly that the above predictions worked in both tissue and ctDNA sequencing with the advantages of liquid biopsy, including noninvasiveness, repeatability, higher accessibility, and reduced deviations caused by tumor heterogeneity and applicable to more advanced patients. All the above PTPRD phosphatase-mut predictions were independent of the TMB, PD-L1 expression, or TP53/KRAS/EGFR mutations. Whether combination of PTPRD mutation and TMB/PD-L1 expression will bring higher efficiency is worthy of further investigation. Moreover, variant interpreters were more over single tools but not reflections of real influences, plasmid containing specific alteration was suggested to transfected into lung cancer cells to validate the predictions of specific mutations’ effects on PTPRD biological functions.

To explain why PTPRD mutations were linked to favorable ICIs outcomes, a potential underlying mechanism might be PTPRD regulating the TGF-β pathway and immune checkpoint molecules, T effectors, and IFN-γ effectors, finally influencing CD8+ T cell infiltration via its phosphatase activity and making TIME “hot.” Here, we provide novel clues about the associations between PTPRD and TIME, while molecular biological experiments were still warrant to verify the specific mechanism.

We also found that PTPRD was linked to OS in melanoma patients treated with ICIs and in bladder cancer patients, there was also a such tendency (*p*=0.062). Consistent with our findings, a recent study reported that the interaction of PTPRD (referred to WT) with its ligand LRFN4 was related to a poor response to Atezolizumab in bladder cancer s[31]. Thus, whether PTPRD mutations were a pan-cancer biomarker for ICIs was worthy of further investigations. Additionally, prospective trials incorporating tissue or ctDNA PTPRD phosphatase-mut as a biomarker are worth conducting both in NSCLC and other cancer types. We are planning to validate these findings prospectively in several coming soon randomized phase II/III study of a PD-(L)1 antibody in multiple cancer types.

This study had several limitations. First, it was a retrospective analysis based on our own cohort and 7 public cohorts. Prospective studies were requested for further verification. Second, a certain degree of research heterogeneity existed. Different cohorts used different strategies, such as the monotherapy of anti-PD-(L)1 or combination with anti-CTLA-4. The predictive efficiency of PTPRD in different drugs requires further investigation. Third, molecular biological experiments are still warranted to validate how specific PTPRD mutations influence immune-related pathways. Last but not least, whether ctDNA PTPRD mutations were consistent with tissue was still unclear. In our previous study, we had proved that ctDNA EGFR mutations were consistent (although not totally) with tissue EGFR mutations and could predict the outcomes of response to EGFR TKI [48]. Hence, like EGFR mutations, ctDNA PTPRD mutations were also supposed to be consistent with tissue mutations. In our present study, the mutation frequencies were similar (both about 10%) across tissue and ctDNA cohorts and PTPRD mutations were associated with PFS and ORR in both tissue and ctDNA cohorts; PTPRD mutations in ctDNA were consequently inferred to be consistent with tumor tissue. Nevertheless, paired tissue and ctDNA samples are expected to been sequenced to verify that in the future.

## Conclusion

To conclude, our study demonstrated that both tissue and ctDNA PTPRD phosphatase-mut could serve as a prognostic biomarker predicting the ORR and PFS. Tissue PTPRD phosphatase-mut was also a prognostic biomarker for OS. Additionally, PTPRD phosphatase-mut could also select benefiting patients between ICIs and chemotherapy. Further prospective trials are warranted.

## Supplementary Information

**Additional file 1: Table S1.** Patient characteristics in NCC cohort. **Table S2.** Details of 8 NSCLC cohorts analyzed in this study. **Table S3.** Prediction of each PTPRD mutations based on PROVEAN. **Table S4.** GSEA of TCGA-LUSC cohort.

**Additional file 2: Figure S1.** PTPRD mutation was not a prognostic biomarker for ICIs in squamous lung cancers. a-c PFS of PTPRD mutation/WT group squamous lung cancer in validation cohort-1 (a), validation cohort-2 (b), MSKCC-240 cohort (c). d OS of PTPRD mutation/WT group squamous lung cancer in MSKCC-350 cohort. e-g PFS of PTPRD mutation/WT group squamous lung cancer in validation cohort-3 (e), OAK (f) and POPLAR (g) cohort. h-j OS of PTPRD mutation/WT group of squamous lung cancers in validation cohort-3 (h), OAK (i) and POPLAR (j) cohort. **Figure S2.** Predicting PFS efficiency of PTPRD mutation in 4 tissue cohorts. a-b PFS of PTPRD mutation/WT group of NSCLC (a) or ns-NSCLC (b) in Miao & N.Rizvi cohort. c-d PFS of PTPRD mutation/WT group NSCLC (c) or ns-NSCLC (d) in Hellmann cohort. e-f PFS of PTPRD mutation/WT group of NSCC (e) or ns-NSCLC (f) in MSKCC-240 cohort. **Figure S3.** Predicting OS efficiency of PTPRD mutation in 2 tissue cohorts. a-b Overall survivals of PTPRD mutation/WT group of NSCLC (a) or ns-NSCLC (b) in MSKCC-350. c-d Overall survivals of PTPRD mutation/WT group of NSCLC (c) or ns-NSCLC (d) in Miao cohort. **Figure S4.** Predicting prognosis efficiency of ctDNA PTPRD mutation in OAK/POPLAR cohort. a-b PFS of PTPRD mutation/WT group of NSCLC in OAK (a) or POPLAR (b) cohort. c-d PFS of PTPRD mutation/WT group of ns-NSCLC in OAK (c) or POPLAR (d) cohort. e-f OS of PTPRD mutation/WT group of NSCLC in OAK (e) or POPLAR (f) cohort. g-h OS of PTPRD mutation/WT group of ns-NSCLC in OAK (g) or POPLAR (h) cohort. **Figure S5.** Predicting prognosis efficiency of PTPRD other-mut. a-b PFS of phosphatase-mut (a) or WT (b) vs other-mut in ns-NSCLC of the discovery cohort. c-d PFS of phosphatase-mut (c) or WT (d) vs other-mut in ns-NSCLC of validation cohort-1. e-f OS of phosphatase-mut (e) or WT (f) vs other-mut in ns-NSCLC of validation cohort-2. g-h PFS of phosphatase-mut (g) or WT (h) vs other-mut in ns-NSCLC of a merged cohort of validation cohort-3. i-j OS of phosphatase-mut (i) or WT (j) vs other-mut in ns-NSCLC of validation cohort-3. **Figure S6.** Predicting prognosis efficiency of PTPRD different mutation types in tissue cohorts. a-c PFS of WT vs phosphatase-mut (a), other-mut vs WT (b) and phosphatase-mut vs other-mut (c) in ns-NSCLC in the MSKCC-240 cohort. d-f PFS of WT vs phosphatase-mut (d), other-mut vs WT (e) and phosphatase-mut vs other-mut (f) in ns-NSCLC in the Hellmann cohort. g-i PFS of WT vs phosphatase-mut (g), other-mut vs WT (h) and phosphatase-mut vs other-mut (i) in ns-NSCLC in the Miao & N.Rizvi cohort. j-l OS of WT vs phosphatase-mut (j), other-mut vs WT (k) and phosphatase-mut vs other-mut (l) in ns-NSCLC in the MSKCC-350 cohort. m-o OS of WT vs phosphatase-mut (m), other-mut vs WT (n) and phosphatase-mut vs other-mut (o) in ns-NSCLC in the Miao cohort. **Figure S7.** Predicting prognosis efficiency of different PTPRD mutation types in ctDNA cohorts.a-c PFS of WT vs phosphatase-mut (a), other-mut vs WT (b) and phosphatase-mut vs other-mut (c) in ns-NSCLC in the OAK cohort. d-f PFS of WT vs phosphatase-mut (d), other-mut vs WT (e) and phosphatase-mut vs other-mut (f) in ns-NSCLC in the POPLAR cohort. g-i OS of WT vs phosphatase-mut (g), other-mut vs WT (h) and phosphatase-mut vs other-mut (i) in ns-NSCLC in the OAK cohort. j-l OS of WT vs phosphatase-mut (j), other-mut vs WT (k) and phosphatase-mut vs other-mut (l) in ns-NSCLC in the POPLAR cohort. **Figure S8.** PTPRD phosphatase-mut predicting prognosis efficiency was independent on TP53/EGFR/KRAS mutation. a-d Interaction plot of PTPRD and TP53, EGFR, KRAS, STK11, KEAP1 in WES cohort (a), MSKCC-240 and MSKCC-350 cohort (b), OAK/POPLAR cohort (c) and TCGA-LUAD cohort (d). e-i HR forest plot of TP53/EGFR/KRAS in ns-NSCLC of discover cohort (e), validation cohort-1(f), validation cohort-2(g) and validation cohort-3 (h-i). **Figure S9.** PTPRD is not a prognosis biomarker for surgery and adjuvant chemotherapy in lung adenocarcinomas. a-b PFS of WT vs mutation (a) or phosphatase-mut (b) in TCGA-LUAD cohort. c PFS of PTPRD expression high vs low in TCGA-LUAD. d-e OS of WT vs mutation (d) or phosphatase-mut (e) in TCGA-LUAD cohort. f OS of PTPRD expression high vs low in TCGA-LUAD. **Figure S10.** PTPRD mutation predicting prognosis efficiency in other cancer types.a-f PTPRD mutations predicting OS efficiency in melanoma (a), bladder cancer (b), esophagastric cancer (c), head and neck cancer (d), colorectal cancer (e) and cancer of unknown primary (f) of NG1661 cohort.

## Data Availability

The validation cohorts (including N.Rizvi cohort, Hellmann cohort, Miao cohort, MSKCC-240, and NG1661 cohort) used in this study were publicly available as described in the “Methods” section. Due to the regulations of the institution, individual-level sequencing and detailed clinical data of NCC cohort of this study cannot be uploaded to the public repository, but available from the corresponding author on reasonable request.

## Abbreviations

bTMB: Blood tumor mutation burden
CNVs: Copy number variations
CR: Complete response
CTC: Circulation tumor cells
ctDNA: Circulating tumor deoxyribonucleic acid
CXCL8: C–X–C motif chemokine ligand 8
ECOG: Eastern Cooperative Oncology Group
EGFR: Epidermal growth factor receptor
GSEA: Gene set enrichment analysis
HR: Hazard ratio
ICIs: Immune checkpoint inhibitors
IFN-γ: Interferon-γ
LUAD: Lung adenocarcinoma
LUSC: Lung squamous cell carcinoma
NSCLC: Non-small cell lung cancer
ORR: Objective response rate
OS: Overall survivals
PD: Progressive disease
PD-1: Program death 1
PD-L1: Program death ligand 1
PFS: Progression-free survivals
PR: Partial response
PTKs: Protein tyrosine kinase
PTPRA: Protein tyrosine phosphatase receptor type A
PTPRC: Protein tyrosine phosphatase receptor type C
PTPRD: Protein tyrosine phosphatase receptor type D
PTPRJ: Protein tyrosine phosphatase receptor type J
PTPRs: Protein tyrosine phosphatase receptor type
SD: Stable disease
TCGA: The Cancer Genome Atlas
TCR: T cell antigen receptor
TGF-β: Transform growth factor β
TILs: Tumor infiltrated lymphocytes
TIME: Tumor immune microenvironment
tTMB: Tissue tumor mutation burden
WES: Whole exon sequencing
WT: Wild type
