# Supplementary material for: Identification and validation of tissue or ctDNA PTPRD phosphatase domain deleterious mutations as prognostic and predictive biomarkers for immune checkpoint inhibitors in non-squamous NSCLC
Source: BMC Med. 2021 Oct 7;19:239. doi: 10.1186/s12916-021-02075-5 (PMC8496052; doi:10.1186/s12916-021-02075-5)
Supplement: Supplementary file 1 — Additional file 1: Table S1. Patient characteristics in NCC cohort. Table S2. Details of 8 NSCLC cohorts analyzed in this study. Table S3. Prediction of each PTPRD mutations based on PROVEAN. Table S4. GSEA of TCGA-LUSC cohort. [file 12916_2021_2075_MOESM1_ESM.pdf]

Additional file 1: Table S1.

Characteristics		No.	%
No. of patients		73	100
**Age**			
	≥60	25	34.25
	<60	48	65.75
**Gender**			
	Female	22	30.14
	Male	51	69.86
**Histology**			
	Non-squamous	49	67.12
	Squamous	24	32.88
**Smoking**			
	Never	37	50.68
	Ever	6	8.22
	Current	30	41.10
**Ecog**			
	0	18	24.66
	1	55	75.34
**Metastasis**			
	Metastasic sites ≥3	26	35.62
	Metastasic sites <3	47	64.38
**LDH**			
	≥250 U/L	34	46.58
	< 250 U/L	39	53.42
**Treatment**			
	Anti-PD1	54	73.97
	Anti-PD-L1	19	19.00
**Lines of therapy**			
	Second	39	53.42
	Third or subsequent	34	46.58
**PD-L1 expression**			
	< 1%	35	47.95
	1-49%	11	15.07
	>50%	3	4.11
	unknown	23	31.51
**Best overall response**			
	CR	1	1.37
	PR	13	17.81
	SD	19	26.03
	PD	40	54.79
**Driver mutation**			
	EGFR	9	12.33
	KRAS	7	9.59
	TP53	36	49.32
**TMB**			
	≥10	18	24.66
	< 10	55	75.34

Additional file 1: Table S2.Details of 8 NSCLC cohorts analyzed in this study.

cohort	cases	sequencing	source	drugs	PFS	OS	responses
NCC	73	WES	tissue	anti-PD-(L)1	yes	NA	ORR
N.Rizvi	34*	WES	tissue	anti-PD-1	yes	NA	ORR
Miao	56	WES	tissue	anti-PD-(L)1<br>+anti-CTLA-4	yes	yes	ORR
Hellmann	75	WES	tissue	anti-PD-(L)1<br>+anti-CTLA-4	yes	NA	ORR
MSKCC-240	240	IMAPCT<br>341/410/468	tissue	anti-PD-(L)1	yes	NA	DCB
MSKCC-350	350	IMPACT<br>341/410/468	tissue	anti-PD-(L)1	NA	yes	NA
OAK	408	Foundation One	ctDNA	anti-PD-L1	yes	yes	ORR
POPLAR	134	Foundation One	ctDNA	anti-PD-L1	yes	yes	ORR

Note: 28 patients with PFS data were shared by N.Rizvi cohort and Miao cohort. Hence, when analyzing PFS, these 2 cohorts were merged as Miao & N.Rizvi cohort. Besides, MSKCC-240 and MSKCC-350 also shared some patients, but MSKCC-240 cohort only contained PFS data while MSKCCCK-350 only contained PFS data. So, MSKCC-240 and MSKCC-350 cohort were still analyzed separately.

Additional file 1: Table S3.

Variant	PROVEAN score	Prediction	Variant	PROVEAN score	Prediction	Variant	PROVEAN score	Prediction
discovery cohort			MSKCC-350 cohort			OAK&POPLAR cohort		
V115I	-0.506	Neutral	Y551F	-1.808	Neutral	D32Y	-5.911	Deleterious
R1798Q	-3.485	Deleterious	V635L	-0.505	Neutral	A143T	-2.678	Deleterious
R1730L	-6.204	Deleterious	V1686L	-2.765	Deleterious	A1664D	-5.526	Deleterious
R141H	-2.427	Neutral	V1063L	-2.299	Neutral	A996P	-3.878	Deleterious
Hellmann cohort			T706K	-4.103	Deleterious	S896L	-2.696	Deleterious
K909N	-2.3	Neutral	T376R	-4.262	Deleterious	Q1422P	-5.524	Deleterious
P867T	-1.892	Neutral	T1738S	-3.556	Deleterious	P986Q	-4.309	Deleterious
L674F	-2.249	Neutral	T1100K	-2.717	Deleterious	T834I	-3.678	Deleterious
G920V	-6.228	Deleterious	S896L	-2.696	Deleterious	S1244C	-4.754	Deleterious
H1345N	-3.606	Deleterious	S667L	-1.218	Neutral	R141H	-2.427	Neutral
P1257L	-6.024	Deleterious	Y1191C	-8.505	Deleterious	A1133S	0.95	Neutral
M1731I	-3.371	Deleterious	S1252Q	-1.304	Neutral	L1014Q	-1.379	Neutral
Miao & N.Rizvi cohort			R6M	-0.045	Neutral	A213P	-2.935	Deleterious
G1057V	-2.143	Neutral	R1674S	-5.5	Deleterious	H660Y	-1.741	Neutral
A773P	-2.086	Neutral	R1536H	-4.39	Deleterious	V736L	-0.288	Neutral
T487K	-4.966	Deleterious	Q425K	-2.461	Neutral	G1094V	-7.167	Deleterious
R1674C	-7.378	Deleterious	Q485K	-0.788	Neutral	A1846S	-2.706	Deleterious
R1793S	-5.147	Deleterious	P519T	-6.669	Deleterious	C45F	-8.014	Deleterious
C1562Y	-9.964	Deleterious	P1839R	-7.831	Deleterious	R237T	-4.488	Deleterious
MSKCC-240 cohort			P1525T	-7.215	Deleterious	E1062V	-2.76	Deleterious
S1252Q	-1.304	Neutral	P1520T	-7.237	Deleterious	R1323L	-6.333	Deleterious
E138Q	-2.208	Neutral	M282I	-3.258	Deleterious	F1534V	-6.362	Deleterious
F850L	-0.116	Neutral	M1864K	-5.418	Deleterious	A1533D	-3.916	Deleterious
T930N	-0.305	Neutral	L984F	-3.029	Deleterious	R561Q	-0.721	Neutral
T869N	-1.061	Neutral	L673V	-0.204	Neutral	G920V	-6.228	Deleterious
V1063L	-2.299	Neutral	L1349F	-3.564	Deleterious	G272R	-6.694	Deleterious
V1686L	-2.765	Deleterious	G761C	-5.375	Deleterious	D281H	-4.049	Deleterious
A1413D	-5.389	Deleterious	G1835A	-5.231	Deleterious	T342K	-4.396	Deleterious
G1835A	-5.231	Deleterious	G1825R	-6.962	Deleterious	Y1910N	-7.204	Deleterious
P1520T	-7.237	Deleterious	G1418W	-7.543	Deleterious	R1559G	-6.328	Deleterious
P519T	-6.669	Deleterious	G1213E	-5.408	Deleterious	R1496L	-6.312	Deleterious
P1338T	-5.937	Deleterious	G1152V	-5.979	Deleterious	D1600E	-3.146	Deleterious
G1418W	-7.543	Deleterious	F850L	-0.116	Neutral	W1729L	-11.516	Deleterious
G1152V	-5.979	Deleterious	E698Q	-1.418	Neutral	R1177C	-4.158	Deleterious
G1152V	-5.979	Deleterious	E21Q	-1.064	Neutral	P609S	-4.047	Deleterious
T1100K	-2.717	Deleterious	E1761V	-5.968	Deleterious	G514A	-5.155	Deleterious
D473V	-5.021	Deleterious	E118K	-2.545	Deleterious	A1846S	-2.706	Deleterious
G761C	-5.375	Deleterious	D473Y	-5.276	Deleterious	S1303I	-2.455	Neutral
A1899D	-4.905	Deleterious	D473V	-5.021	Deleterious	T1455K	-5.657	Deleterious
L1349F	-3.564	Deleterious	A1413D	-5.389	Deleterious	D1908Y	-7.207	Deleterious
R1674S	-5.5	Deleterious	TCGA-LUAD			G1122C	-6.35	Deleterious
M1864K	-5.418	Deleterious	A1101S	-1.822	Neutral	R1496Q	-3.502	Deleterious
E118K	-2.545	Deleterious	L1255M	0.15	Neutral	A336G	-3.251	Deleterious
V1389G	-6.606	Deleterious	E1078Q	-0.825	Neutral	P326R	-7.664	Deleterious
			S1179T	-1.557	Neutral	G382V	-7.206	Deleterious
			S1299T	-1.011	Neutral	C1615F	-9.295	Deleterious
			Y1182F	-1.476	Neutral	N1023I	-7.261	Deleterious
			E915D	-2.126	Neutral	K439N	-1.378	Neutral
			A381S	-0.321	Neutral	Q193K	-3.082	Deleterious
			L790I	-1.568	Neutral	C1615F	-9.295	Deleterious
			Q67E	-1.803	Neutral	R857L	-5.06	Deleterious
			R123S	0.793	Neutral	P403L	-7.801	Deleterious
			A1846T	-3.611	Deleterious	Q1018K	-1.811	Neutral
			G1638A	-4.797	Deleterious	H660Y	-1.741	Neutral
			L1532V	-2.643	Deleterious	T1483R	-5.235	Deleterious
			A100D	-4.411	Deleterious	S1395C	-4.403	Deleterious
			Q1718H	-4.416	Deleterious	P1839A	-6.969	Deleterious
			R1730W	-7.082	Deleterious	S182C	-0.29	Neutral
			D1344N	-2.672	Deleterious	E1786G	-6.006	Deleterious
			W1038R	-12.688	Deleterious	T706N	-3.439	Deleterious
			P1249T	-4.48	Deleterious	D154Y	-6.188	Deleterious
			P1318T	-7.342	Deleterious	T477S	-3.054	Deleterious
			R1181P	-4.625	Deleterious	R684L	-2.879	Deleterious
			D500Y	-7.173	Deleterious	P1530L	-8.563	Deleterious
			D275N	-3.694	Deleterious	E21K	-1.259	Neutral
			L925H	-4.777	Deleterious	T988N	-3.667	Deleterious
			Q316R	-3.255	Deleterious	M1731I	-3.371	Deleterious
			R174L	-4.783	Deleterious	F1011L	-1.187	Neutral
			R733H	-2.987	Deleterious	G789V	-4.204	Deleterious
			S180K	-2.71	Deleterious	L1255Q	-1.163	Neutral
			T804K	-4.775	Deleterious	P1433S	-6.285	Deleterious
			Y488D	-8.824	Deleterious	P759R	-2.723	Deleterious
						D371H	-4.309	Deleterious
						T158S	-0.762	Neutral
						L146F	-2.86	Deleterious
						S504L	-5.161	Deleterious
						S504T	-2.614	Deleterious
						S927L	-0.256	Neutral
						Q1368E	-2.823	Deleterious
						T301P	-5.151	Deleterious
						L435V	-0.91	Neutral
						A426E	-4.054	Deleterious
						E1902V	-5.795	Deleterious
						P421L	-8.8	Deleterious
						A1295V	-1.558	Neutral
						P792A	-5.396	Deleterious

Additional file 1: Table S4.

NAME	SIZE	ES	NES	NOM p-value	FDR q-value	FWER p-value
HALLMARK_UV_RESPONSE_DN	134	-0.424	-1.359	0.031	0.376	0.469
HALLMARK_HEDGEHOG_SIGNALING	34	-0.476	-1.421	0.047	0.432	0.339
HALLMARK_EPITHELIAL_MESENCHYMAL_TRANSITION	194	-0.565	-1.273	0.092	0.522	0.601
HALLMARK_MYOGENESIS	199	-0.323	-1.264	0.094	0.420	0.616
HALLMARK_INTERFERON_ALPHA_RESPONSE	90	0.501	1.405	0.113	0.663	0.349
HALLMARK_OXIDATIVE_PHOSPHORYLATION	179	0.530	1.260	0.129	1.000	0.597
HALLMARK_REACTIVE_OXYGEN_SPECIES_PATHWAY	45	-0.474	-1.163	0.209	0.684	0.696
HALLMARK_KRAS_SIGNALING_DN	188	0.172	1.130	0.218	1.000	0.716
HALLMARK_PANCREAS_BETA_CELLS	38	0.346	1.210	0.228	1.000	0.651
HALLMARK_PEROXISOME	100	0.309	1.099	0.268	0.901	0.750
HALLMARK_MYC_TARGETS_V2	57	0.418	1.177	0.274	0.996	0.679
HALLMARK_DNA_REPAIR	138	0.387	1.128	0.284	0.896	0.718
HALLMARK_MITOTIC_SPINDLE	194	-0.297	-1.118	0.346	0.742	0.733
HALLMARK_INTERFERON_GAMMA_RESPONSE	190	0.356	1.083	0.374	0.856	0.768
HALLMARK_IL6_JAK_STAT3_SIGNALING	86	0.391	1.063	0.381	0.841	0.789
HALLMARK_SPERMATOGENESIS	130	0.144	1.024	0.415	0.899	0.827
HALLMARK_XENOBIOTIC_METABOLISM	198	-0.280	-1.027	0.422	0.995	0.811
HALLMARK_MYC_TARGETS_V1	189	0.478	1.010	0.464	0.873	0.838
HALLMARK_PROTEIN_SECRETION	95	-0.338	-1.002	0.469	0.972	0.834
HALLMARK_E2F_TARGETS	186	0.345	0.963	0.507	0.893	0.874
HALLMARK_ANGIOGENESIS	36	-0.416	-0.976	0.525	0.960	0.863
HALLMARK_ALLOGRAFT_REJECTION	189	0.340	0.975	0.530	0.921	0.863
HALLMARK_HYPOXIA	188	-0.329	-0.974	0.538	0.871	0.863
HALLMARK_HEME_METABOLISM	187	-0.198	-0.945	0.592	0.890	0.887
HALLMARK_TNFA_SIGNALING_VIA_NFKB	194	0.339	0.913	0.632	0.773	0.912
HALLMARK_UV_RESPONSE_UP	152	0.341	0.944	0.640	0.890	0.884
HALLMARK_PI3K_AKT_MTOR_SIGNALING	101	0.289	0.929	0.642	0.822	0.901
HALLMARK_ADIPOGENESIS	185	0.267	0.925	0.654	0.786	0.905
HALLMARK_COMPLEMENT	193	0.268	0.898	0.656	0.771	0.918
HALLMARK_ESTROGEN_RESPONSE_LATE	196	0.298	0.938	0.683	0.852	0.895
HALLMARK_APICAL_JUNCTION	193	-0.263	-0.879	0.702	1.000	0.922
HALLMARK_P53_PATHWAY	193	0.277	0.896	0.720	0.737	0.918
HALLMARK_CHOLESTEROL_HOMEOSTASIS	73	-0.297	-0.855	0.738	0.947	0.940
HALLMARK_COAGULATION	136	-0.260	-0.861	0.759	0.999	0.936
HALLMARK_TGF_BETA_SIGNALING	54	-0.306	-0.837	0.764	0.876	0.951
HALLMARK_FATTY_ACID_METABOLISM	155	-0.260	-0.837	0.794	0.934	0.951
HALLMARK_G2M_CHECKPOINT	183	0.281	0.721	0.835	0.952	0.992
HALLMARK_NOTCH_SIGNALING	32	0.263	0.794	0.842	0.967	0.970
HALLMARK_GLYCOLYSIS	192	-0.249	-0.810	0.860	0.895	0.961
HALLMARK_ESTROGEN_RESPONSE_EARLY	190	-0.216	-0.806	0.863	0.855	0.961
HALLMARK_IL2_STAT5_SIGNALING	190	-0.207	-0.786	0.864	0.859	0.967
HALLMARK_UNFOLDED_PROTEIN_RESPONSE	108	0.257	0.791	0.877	0.933	0.973
HALLMARK_MTORC1_SIGNALING	194	-0.306	-0.769	0.882	0.853	0.973
HALLMARK_BILE_ACID_METABOLISM	112	0.139	0.781	0.885	0.919	0.979
HALLMARK_APICAL_SURFACE	42	0.168	0.762	0.896	0.930	0.986
HALLMARK_WNT_BETA_CATENIN_SIGNALING	42	-0.243	-0.721	0.917	0.871	0.985
HALLMARK_ANDROGEN_RESPONSE	95	-0.240	-0.764	0.922	0.823	0.974
HALLMARK_INFLAMMATORY_RESPONSE	197	0.229	0.719	0.923	0.918	0.992
HALLMARK_KRAS_SIGNALING_UP	194	0.193	0.711	0.980	0.899	0.992
HALLMARK_APOPTOSIS	159	0.291	0.758	0.982	0.903	0.986
KEGG_TGF_BETA_SIGNALING_PATHWAY	85	-0.243	-0.786	0.873	0.931	1.000
KEGG_WNT_SIGNALING_PATHWAY	149	-0.221	-0.905	0.639	0.937	1.000
KEGG_VEGF_SIGNALING_PATHWAY	74	0.220	0.841	0.746	1.000	1.000
