# Supplementary figures and images for: Identification and validation of tissue or ctDNA PTPRD phosphatase domain deleterious mutations as prognostic and predictive biomarkers for immune checkpoint inhibitors in non-squamous NSCLC
Source: BMC Med. 2021 Oct 7;19:239. doi: 10.1186/s12916-021-02075-5 (PMC8496052; doi:10.1186/s12916-021-02075-5)

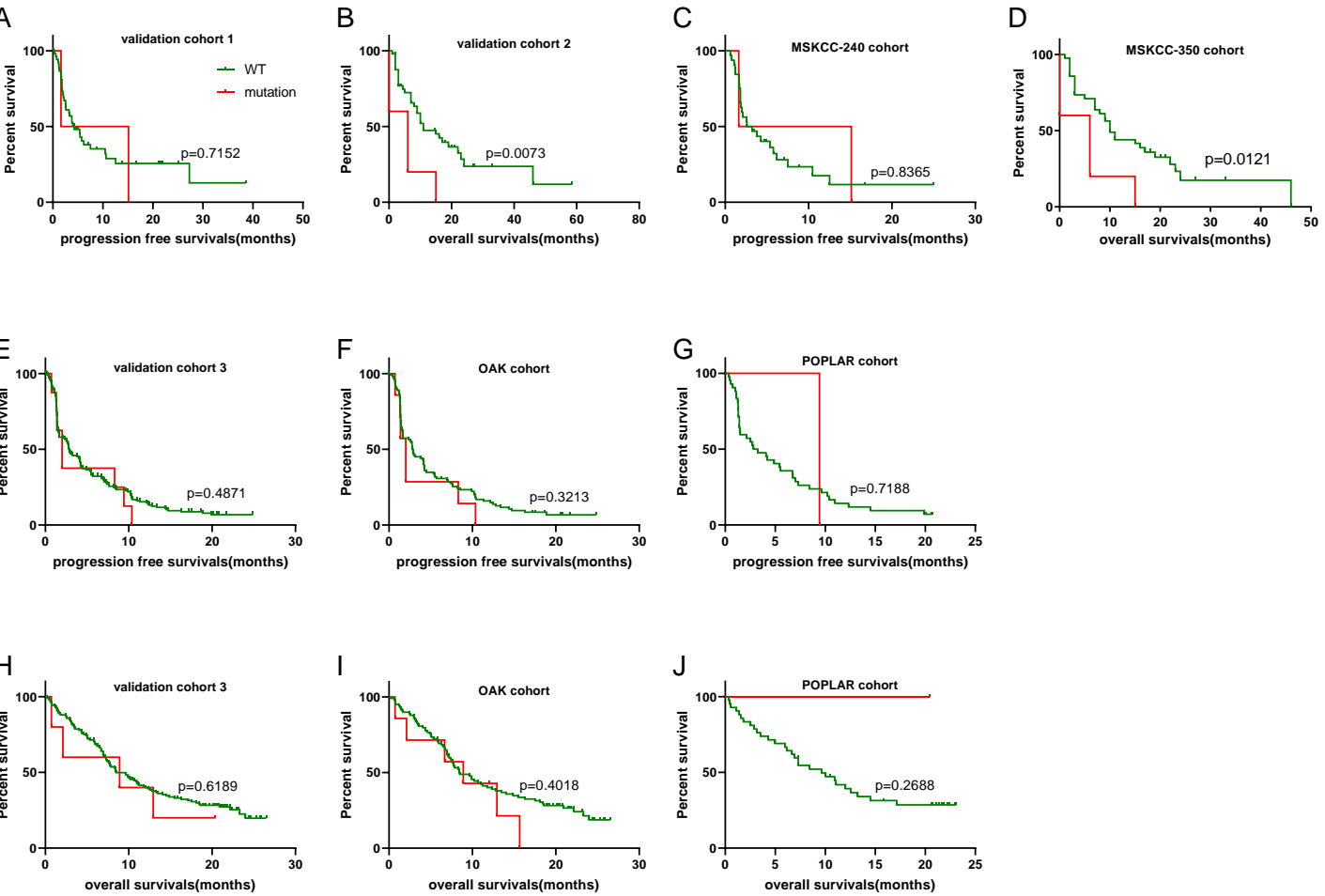

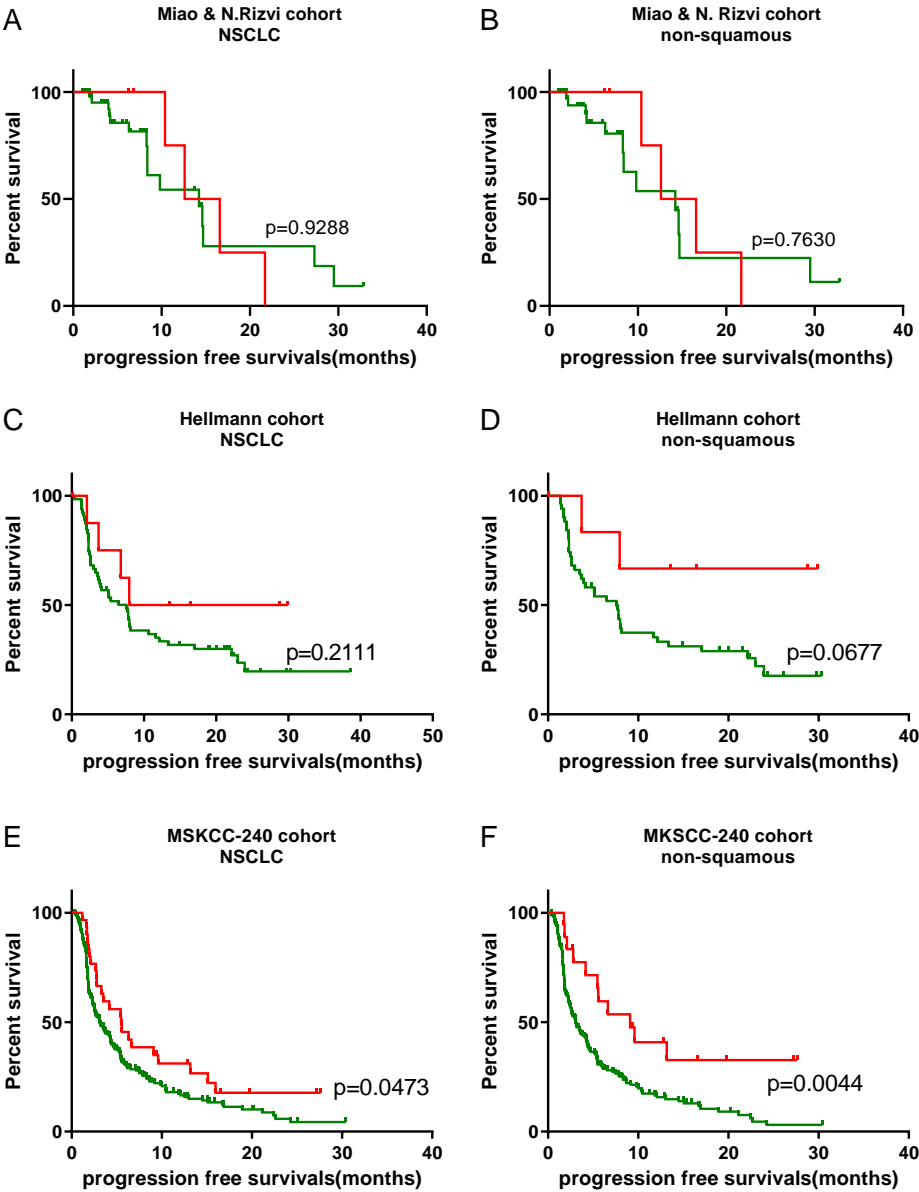

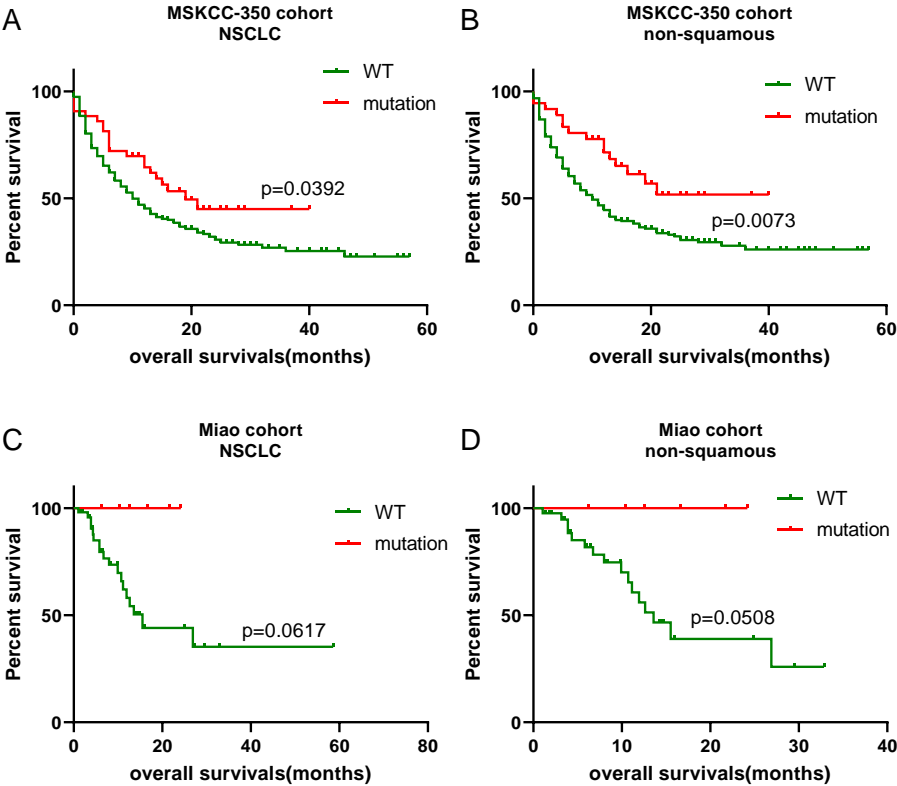

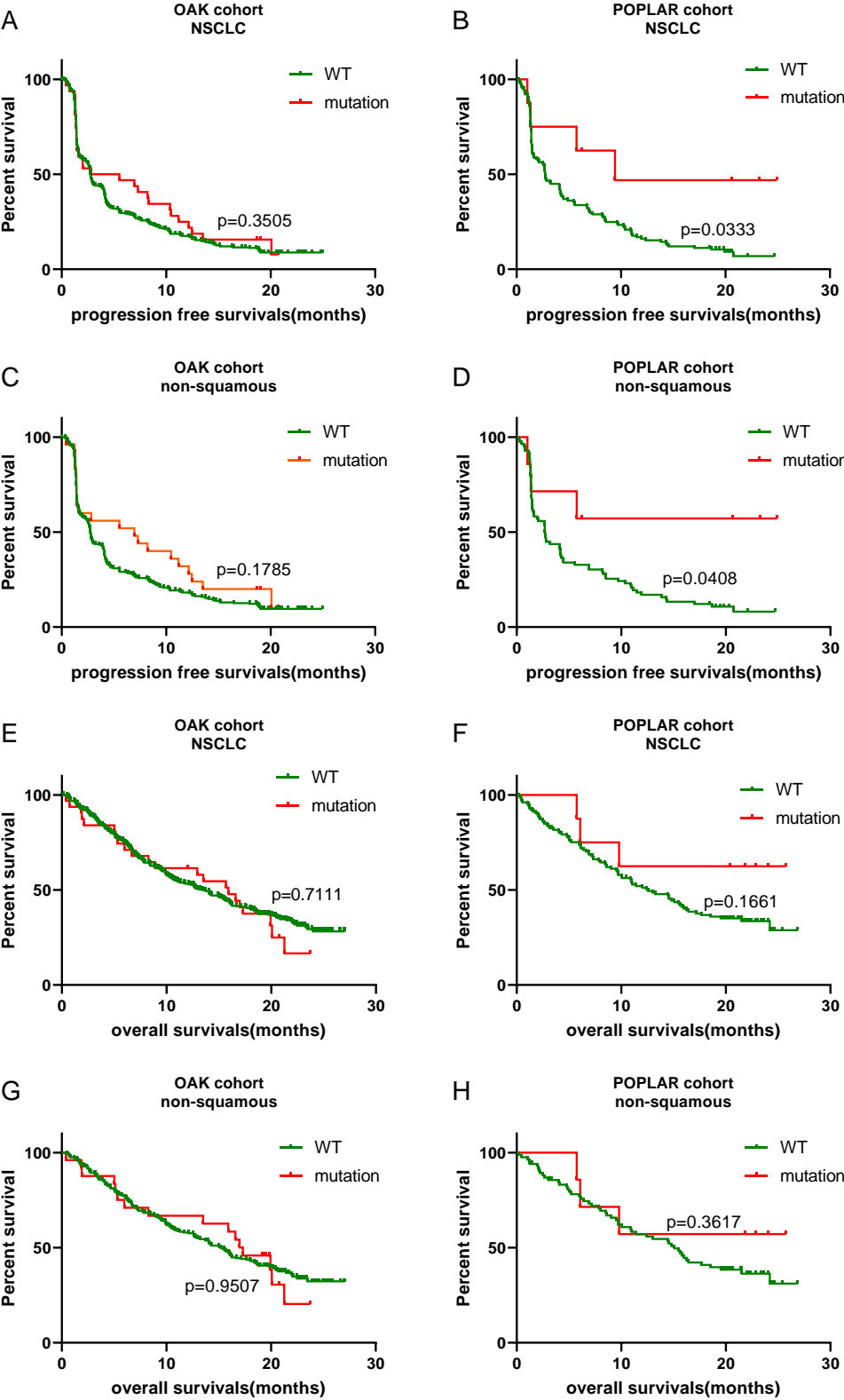

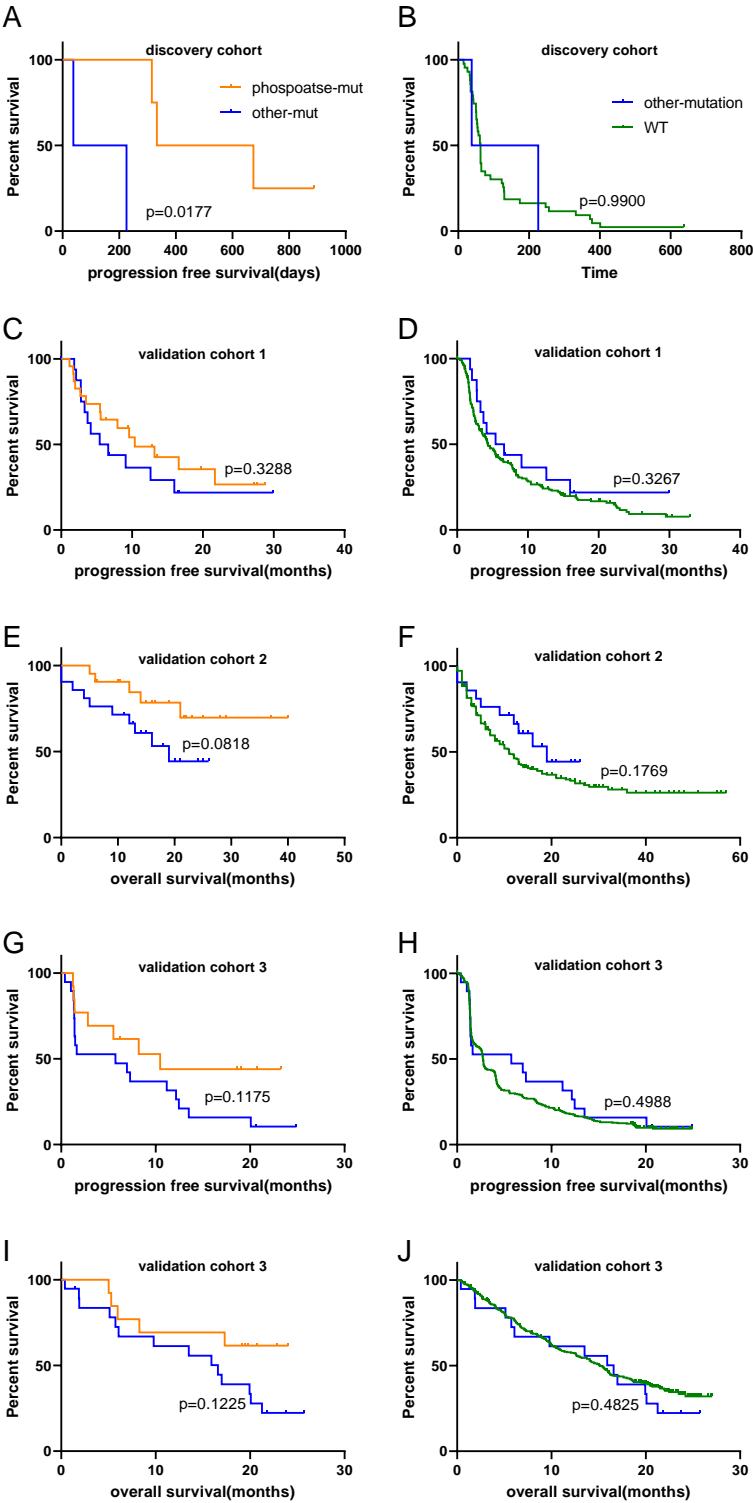

Additional file 2: Figure S6.

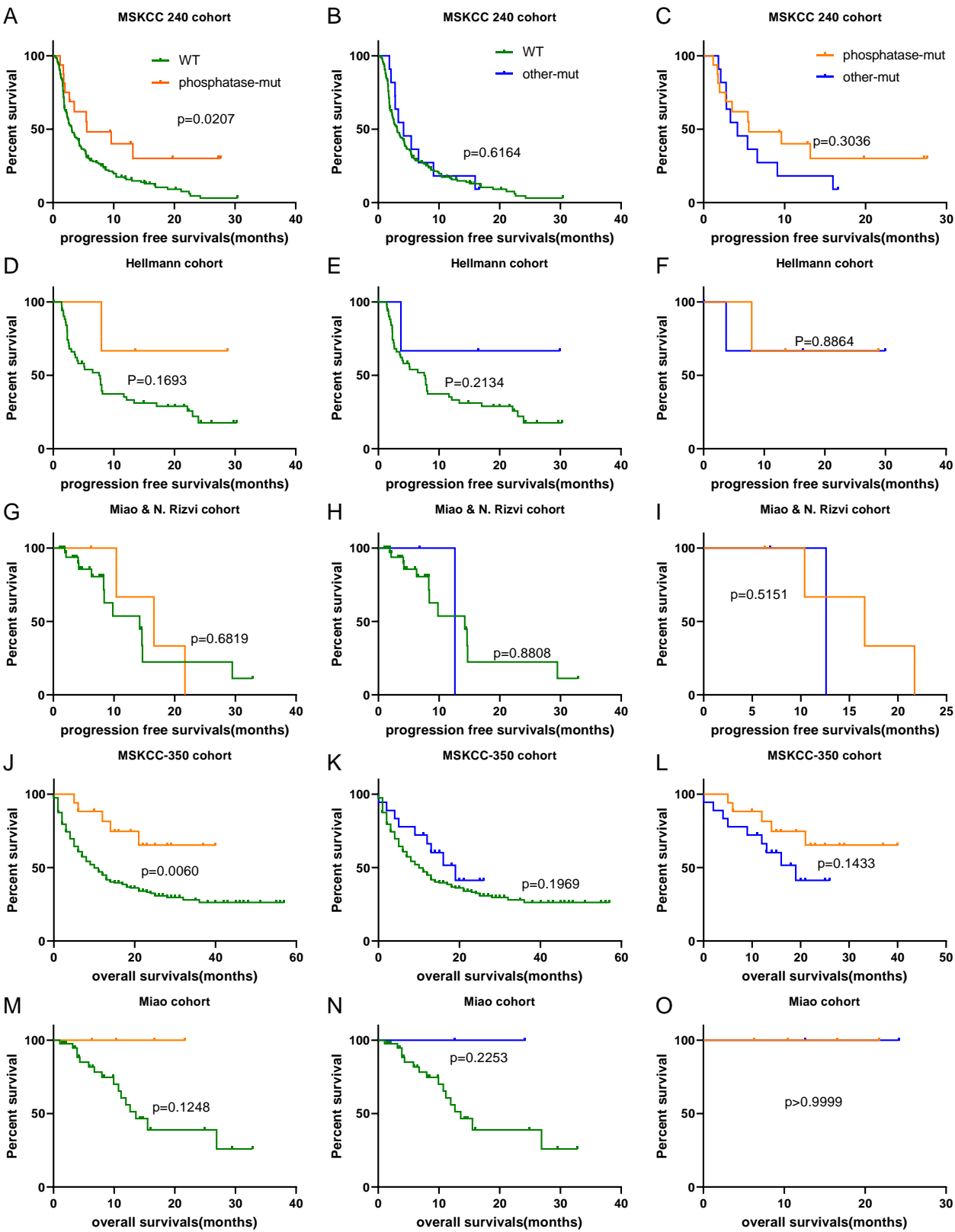

Additional file 2: Figure S7.

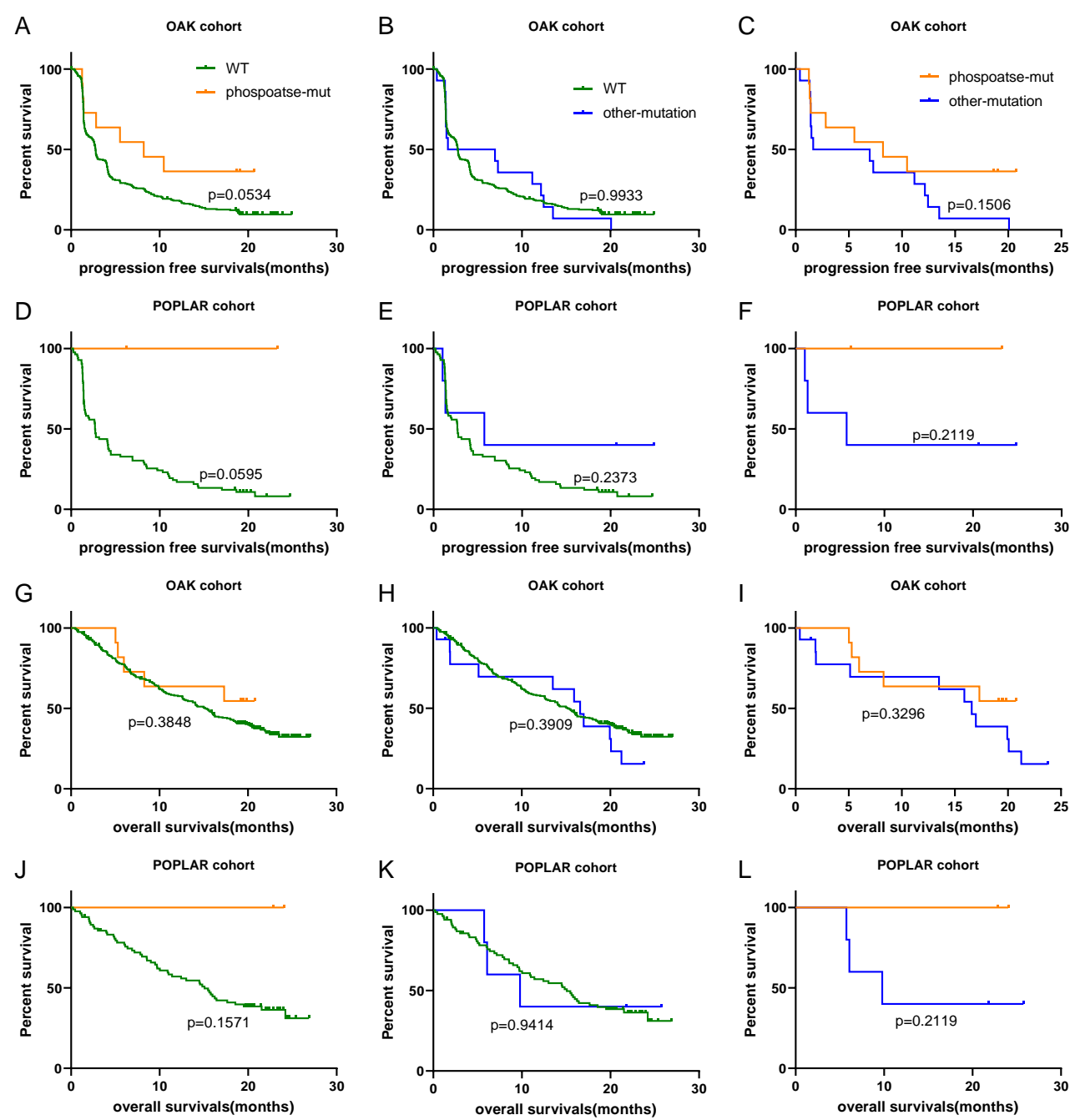

Additional file 2: Figure S8.

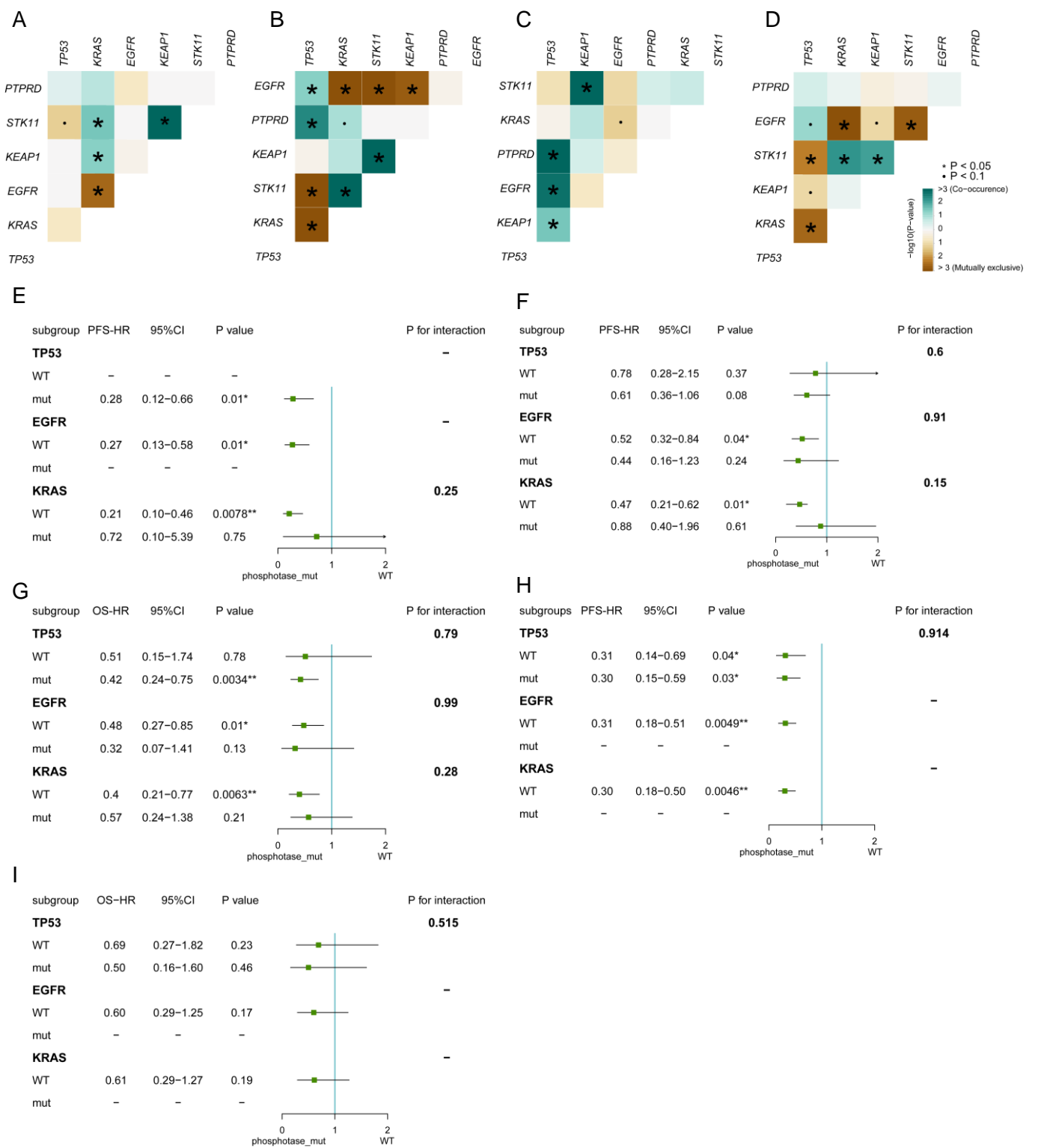

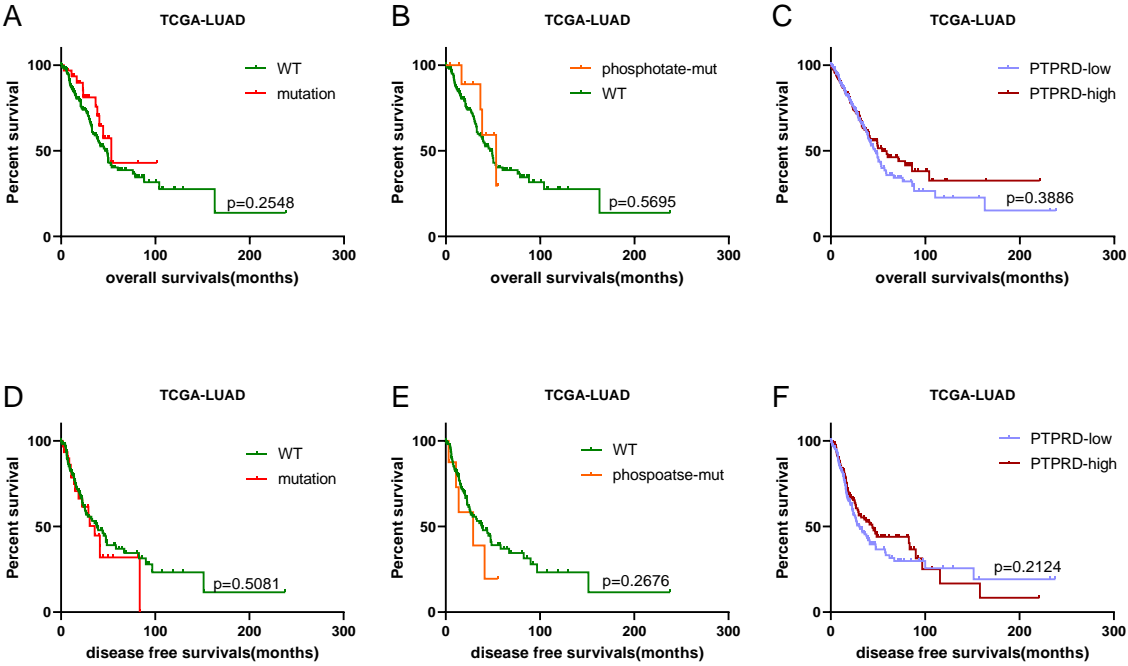

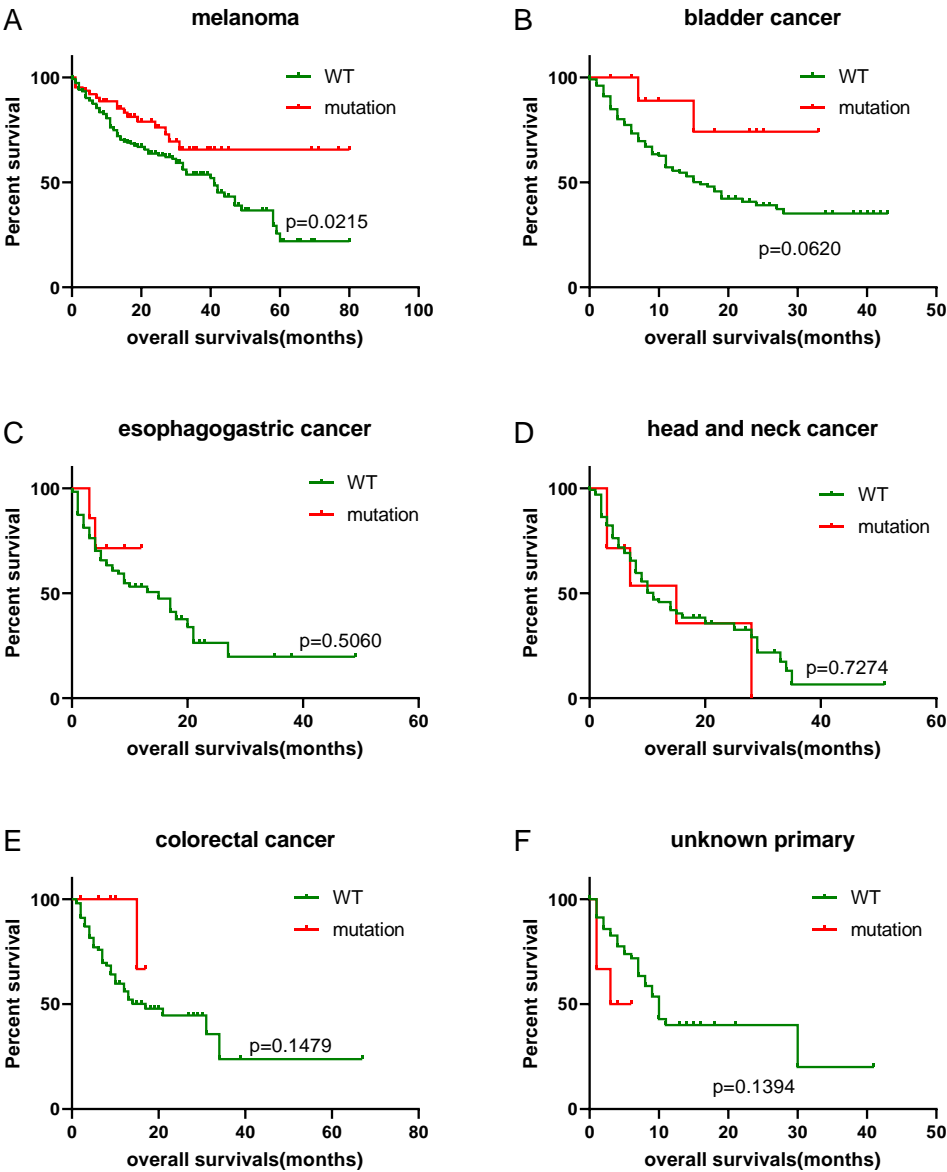

Supplement: Supplementary file 2 — Additional file 2: Figure S1. PTPRD mutation was not a prognostic biomarker for ICIs in squamous lung cancers. a-c PFS of PTPRD mutation/WT group squamous lung cancer in validation cohort-1 (a), validation cohort-2 (b), MSKCC-240 cohort (c). d OS of PTPRD mutation/WT group squamous lung cancer in MSKCC-350 cohort. e-g PFS of PTPRD mutation/WT group squamous lung cancer in validation cohort-3 (e), OAK (f) and POPLAR (g) cohort. h-j OS of PTPRD mutation/WT group of squamous lung cancers in validation cohort-3 (h), OAK (i) and POPLAR (j) cohort. Figure S2. Predicting PFS efficiency of PTPRD mutation in 4 tissue cohorts. a-b PFS of PTPRD mutation/WT group of NSCLC (a) or ns-NSCLC (b) in Miao & N.Rizvi cohort. c-d PFS of PTPRD mutation/WT group NSCLC (c) or ns-NSCLC (d) in Hellmann cohort. e-f PFS of PTPRD mutation/WT group of NSCC (e) or ns-NSCLC (f) in MSKCC-240 cohort. Figure S3. Predicting OS efficiency of PTPRD mutation in 2 tissue cohorts. a-b Overall survivals of PTPRD mutation/WT group of NSCLC (a) or ns-NSCLC (b) in MSKCC-350. c-d Overall survivals of PTPRD mutation/WT group of NSCLC (c) or ns-NSCLC (d) in Miao cohort. Figure S4. Predicting prognosis efficiency of ctDNA PTPRD mutation in OAK/POPLAR cohort. a-b PFS of PTPRD mutation/WT group of NSCLC in OAK (a) or POPLAR (b) cohort. c-d PFS of PTPRD mutation/WT group of ns-NSCLC in OAK (c) or POPLAR (d) cohort. e-f OS of PTPRD mutation/WT group of NSCLC in OAK (e) or POPLAR (f) cohort. g-h OS of PTPRD mutation/WT group of ns-NSCLC in OAK (g) or POPLAR (h) cohort. Figure S5. Predicting prognosis efficiency of PTPRD other-mut. a-b PFS of phosphatase-mut (a) or WT (b) vs other-mut in ns-NSCLC of the discovery cohort. c-d PFS of phosphatase-mut (c) or WT (d) vs other-mut in ns-NSCLC of validation cohort-1. e-f OS of phosphatase-mut (e) or WT (f) vs other-mut in ns-NSCLC of validation cohort-2. g-h PFS of phosphatase-mut (g) or WT (h) vs other-mut in ns-NSCLC of a merged cohort of validation cohort-3. i-j OS of phosph [file 12916_2021_2075_MOESM2_ESM.pdf]
